# Genome analysis of a thermophilic exopolysaccharide-producing bacterium - *Geobacillus* sp. WSUCF1

**DOI:** 10.1038/s41598-018-36983-z

**Published:** 2019-02-07

**Authors:** Jia Wang, Kian Mau Goh, David R. Salem, Rajesh K. Sani

**Affiliations:** 1 Department of Chemical and Biological Engineering, South Dakota School of Mines and Technology, Rapid City, SD 57701 USA; 2 Faculty of Science, Universiti Teknologi Malaysia, Skudai, Johor 81300 Malaysia; 3 Department of Materials and Metallurgical Engineering, South Dakota School of Mines and Technology, Rapid City, SD 57701 USA; 4 Composite and Nanocomposite Advanced Manufacturing – Biomaterials Center (CNAM-Bio Center), Rapid City, SD 57701 USA; 5 BuG ReMeDEE Consortium, South Dakota School of Mines and Technology, Rapid City, SD 57701 USA

## Abstract

*Geobacillus* sp. WSUCF1 is a Gram-positive, spore-forming, aerobic and thermophilic bacterium, isolated from a soil sample obtained from a compost facility. Strain WSUCF1 demonstrated EPS producing capability using different sugars as the carbon source. The whole-genome analysis of WSUCF1 was performed to disclose the essential genes correlated with nucleotide sugar precursor biosynthesis, assembly of monosaccharide units, export of the polysaccharide chain, and regulation of EPS production. Both the biosynthesis pathway and export mechanism of EPS were proposed based on functional annotation. Additionally, the genome description of strain WSUCF1 suggests sophisticated systems for its adaptation under thermophilic conditions. The presence of genes associated with CRISPR-Cas system, quorum quenching lactonase, polyketide synthesis and arsenic resistance makes this strain a potential candidate for various applications in biotechnology and biomedicine. The present study indicates that strain WSUCF1 has promise as a thermophilic EPS producer for a broad range of industrial applications. To the best of our knowledge, this is the first report on genome analysis of a thermophilic *Geobacillus* species focusing on its EPS biosynthesis and transportation, which will likely pave the way for both enhanced yield and tailor-made EPS production by thermophilic bacteria.

## Introduction

*Geobacillus* genus bacteria are Gram-positive, rod-shaped, aerobic or facultatively anaerobic, spore-forming thermophiles with an optimum growth temperature range of 55 to 65 °C^1–3^. By the end of September 2018, 20 species have been classified to *Geobacillus* genus with validly published names^4^, and in the NCBI database 85 *Geobacillus* genomes have been sequenced to unveil the genes with different potential biotechnological applications. Over the past century, *Geobacillus* and other thermophilic bacteria have extended our understanding of biochemistry under inhospitable environmental conditions. The *Geobacillus* spp. strains are irreplaceable microbial contributors to the biotechnological industry due to their valuable thermostable lignocellulolytic enzymes and other thermoactive bioproducts^5^.

It is well-acknowledged that the macromolecules produced from thermophiles - such as thermozymes, thermostable proteins, and exopolysaccharides (EPSs) - improve the ability of the cells to withstand harsh environmental factors^6,7^. Exopolymeric substances (proteins, DNA, and EPS) are essential components in biofilms, which are self-produced matrices^8^. EPSs form an immediate environment suitable for cell adhesion, retention of water and nutrients^9^. In addition, EPSs contribute to the improvement of the mechanical stability of biofilms^10^. For thermophiles, EPSs surround the bacterial cells and generate a highly hydrated boundary against desiccation effects under thermophilic conditions^11,12^.

Several *Geobacillus* strains isolated from terrestrial or marine hot springs and shallow hydrothermal vents have been studied for EPS production^13–16^, and *Geobacillus* strains usually have a wide spectrum of carbon source utilization for EPS production^16^. It has been shown that EPSs with antioxidant activity, immunomodulatory effects, and anti-cytotoxic properties can be produced in a short fermentation period by these thermophiles^13,16,17^. Furthermore, the EPSs from *Geobacillus* strains demonstrated unusually high decomposition temperatures compared with those from other thermophilic bacteria^13,16^. The *Geobacillus* species strains can be important contenders as commercially competitive EPS producers.

In previous studies, the motivation for sequencing of *Geobacillus* species strains was to investigate the genes involved in bioremediation, biofuel production, and enzymatic degradation of long-chain molecules and polymeric substrates^18–21^. However, in-depth studies concerning *Geobacillus* genome analysis to elucidate its EPS-producing potential are very limited, resulting in the narrow understanding of the organization of EPS synthetic pathways and export mechanisms within the *Geobacillus* genome. EPS production usually requires a complex regulation network involving multiple communications among various regulatory components. However, the role of regulatory systems remains only partially understood in terms of how the signal molecules may impact thermophilic EPS biosynthesis.

The genome sequencing and annotation can be a potent tool for understanding the collection of genes a microorganism utilizes for biopolymer production. The disclosure of the EPS biosynthetic pathway may pave the way for the further exploration of design space through metabolic and genomic engineering for overproduction and tailor-made EPSs with desired properties. Besides EPS biosynthesis, uncovering the molecular mechanisms of *Geobacillus* sp. strain as a model microorganism will extend understanding of the resistance of thermophiles to high-temperature conditions.

*Geobacillus* sp. strain WSUCF1 is an aerobic spore-forming thermophilic bacterium, isolated from a soil sample collected from a compost facility^22^. In-house analysis elucidated that *Geobacillus* sp. WSUCF1 produced a significant amount of mannan and glucomannan as two types of EPSs with potential uses in pharmaceutical and biomaterial industries. The aim of this study is to use genome annotation to unravel the pathways involved in EPS biosynthesis and transportation of *Geobacillus* sp. WSUCF1. Furthermore, the genes associated with thermophily and other potential biotechnological and biomedical applications are presented.

## Results

### General features of the thermophilic *Geobacillus* sp. WSUCF1 genome

The genome of *Geobacillus* sp. WSUCF1 consists of 3,402,383 base pairs with an average G + C content of 52.21%. It is a high-quality genome with 97.48% genome completeness and 0.32% contamination. The G + C% of tRNA and rRNA sequences are 59.23% and 58.36%, respectively. The genome annotation of the draft genome of strain WSUCF1 using PATRIC genome annotation service demonstrated 4184 coding sequences and of these genes, 82 were RNA genes (Table 1). 2342 protein-coding genes were assigned to 129 pathways, which was 55.98% of the total coding sequences. The gene annotation revealed several completed metabolic pathways in the genome of strain WSUCF1 e.g., glycolysis, gluconeogenesis, tricarboxylic acid (TCA) cycle, pentose-phosphate, and glyoxylate bypass. The biosynthetic pathways for several amino acids, including valine, leucine, isoleucine, phenylalanine, tyrosine, tryptophan and lysine; and metabolic pathways for several vitamins (biotin, thiamine, vitamin B6, riboflavin) were also assigned. The distribution of genes into clusters of orthologous groups (COGs) functional categories is listed in Table 2.

**Table 1:** General features of *Geobacillus* sp. WSUCF1 draft genome.

Feature	*Geobacillus* sp. WSUCF1
Domain	Bacteria
Taxonomy	*Firmicutes*, *Bacilli*, *Bacillales*, *Bacillaceae*, *Geobacillus*
Genome size	3,402,383 bp
G + C content	52.21%
Completeness	97.48%
Contamination	0.32%
Number of coding sequences (CDSs) in PATRIC	4184
Proteins with functional assignments	3224
Hypothetical proteins	960
Proteins with EC number assignments	1116
Proteins with KEGG pathway assignments	875
Genes assigned to COGs	2675
Number of tRNA	75
Number of rRNA	7
G + C content of tRNA	59.23%
G + C content of rRNA	58.36%
N50 value	22601
L50 value	47
CRISPR repeats	42
CRISPR spacer	37
CRISPR array	5

**Table 2:** Number of genes associated with the general cluster of orthologous group (COG) functional categories.

COG code	Number of genes	Percentage	Description
***Cellular processes and signaling***			
D	26	0.62	Cell cycle control, cell division, chromosome partitioning
M	132	3.11	Cell wall/membrane/envelope biogenesis
N	39	0.93	Cell motility
O	81	1.88	Post-translational modification, protein turnover, and chaperones
T	104	2.32	Signal transduction mechanisms
U	23	0.55	Intracellular trafficking, secretion, and vesicular transport
V	39	0.93	Defense mechanisms
W	0	0	Extracellular structures
Y	0	0	Nuclear structure
Z	0	0	Cytoskeleton
***Information storage and processing***			
A	0	0	RNA processing and modification
B	0	0	Chromatin structure and dynamics
J	132	3.15	Translation, ribosomal structure and biogenesis
K	168	4.02	Transcription
L	140	3.35	Replication, recombination and repair
***Metabolism***			
C	162	3.87	Energy production and conversion
E	212	5.07	Amino acid transport and metabolism
F	54	1.29	Nucleotide transport and metabolism
G	155	3.58	Carbohydrate transport and metabolism
H	103	2.44	Coenzyme transport and metabolism
I	85	2.03	Lipid transport and metabolism
P	142	3.27	Inorganic ion transport and metabolism
Q	41	0.93	Secondary metabolites biosynthesis, transport, and catabolism
***Poorly characterized***			
R	225	5.38	General function prediction only
S	637	15.22	Function unknown
–	1509	36.06	Not in COGs

### Stress tolerance

The stress tolerance genes for heat response, oxidative stress response, osmotic stress, acid resistance and carbon starvation found in WSUCF1 genome play an important role for WSUCF1 in proliferating under thermophilic conditions. The WSUCF1 strain has spermidine synthase gene in its genome. In addition, WSUCF1 appears to be capable of uptaking putrescine and spermidine from the environment through the spermidine/putrescine import ABC transporter system (Supplementary Table S1). The presence of the genes related to linear polyamine synthesis and transport is common in the genome of thermophilic bacteria and considered to be associated with the thermophilicity of thermophiles^3,23^. Other genes involved in adaption in a thermophilic environment, including genes encoding PriA helicase and DNA-binding protein HU, were also found in the genome. The heat shock response system in WSUCF1 includes genes encoding heat shock protein GrpE, chaperone proteins DnaK, DnaJ and GroEL, co-chaperone GroES, and heat-inducible transcription repressors. Besides these heat shock proteins, one gene coding for cold shock protein of the Csp family was found in *Geobacillus* sp. WSUCF1 genome.

Oxidative stress is mostly coupled with heat stress^24^. From the genome annotation, WSUCF1 possesses a complex system to protect against the oxidative stress in a thermophilic environment (Supplementary Table S1). A urease system was found in the WSUCF1 genome and it may contribute to internal pH homeostasis to provide resistance in acidic conditions. Several genes encoding proteins involved in adaptation to osmotic stress are also present in the genome of WSUCF1, such as Ca^2+^/H^+^ antiporter and sodium-glucose/galactose cotransporter. Strain WSUCF1 lacks the genes coding for choline-sulfatase involved in the biosynthesis of the osmoprotectant choline. The genes encoding the osmoprotectant-associated transporter OpuD (glycine betaine transporter) were detected in the WSUCF1 genome. Strain WSUCF1 also encodes potassium uptake proteins KtrB, KtrC, KtrD, KefA, KQT and potassium channel protein, but lacks the genes for ectoine transport and biosynthesis. The genes encoding Na^+^/H^+^ antiporter NhaC related to adaptation to alkaline pH were also found in the WSUCF1 genome. The genes coding for sulfate permease involved in sulfate export in the WSUCF1 genome could be helpful for withstanding a high sulfate content environment.

WSUCF1 has genes encoding the enzymes for base excision repair including several DNA glycosylases (Supplementary Table S1). The DNA glycosylases could remove the damaged or altered bases of DNA. The remaining apurinic and apyrimidinic sites could be excised by endonucleases, and the nucleotide pyrophosphatase could excise the phosphate residues. Finally, the DNA polymerases could repair the gap within the genome. The stretch of damaged DNA is cut by UvrC nucleotide excision repair protein and isolated from the intact genome by a helicase UvrD^3^. The genes for DNA mismatch repair were also found, including DNA mismatch repair proteins MutS and MutL, DNA helicases, single-stranded-DNA-specific exonuclease RecJ, single-stranded DNA-binding protein, DNA polymerase III, and NAD-dependent DNA ligase. Several genes involved in the homologous recombination repair pathway, such as genes encoding recombination proteins RecA and RecR, Holliday junction DNA helicases RuvA and RuvB are present in the WSUCF1 genome.

### Features of biotechnological interest

Multiple genes coding for hemicellulases are present in the WSUCF1 genome, including xylan 1,4-β-xylosidase, endo-1,4-β-xylanase, and xylan α-1,2-glucuronosidase. Intact genes including α-amylase were also found in the WSUCF1 genome (Supplementary Table S2), suggesting that WSUCF1 may be able to break down starch. Besides hydrolases, the quorum-quenching lactonase YtnP is responsible for the enzyme-catalyzed *N-*acylhomoserine lactone (autoinducer) degradation, and its gene was detected in the strain WSUCF1 genome. The gene encoding CRISPR-associated endoribonuclease Cas6 is also present which reflects a previous phage infection event, and it could be promising for *in vitro* molecular biology applications. Furthermore, strain WSUCF1 may use type III polyketide synthase BpsA to produce aromatic ketones, and this thermostable polyketide synthase can be appealing as a robust biosynthetic tool. Finally, the *Geobacillus* sp. WSUCF1 genome carries multiple genes involved in arsenic resistance, including genes for arsenate reductase, arsenical resistance protein ACR3, arsenic efflux pump protein, arsenical resistance operon repressor and transcriptional repressor ArsR family. Those arsenic resistance genes indicate that strain WSUCF1 has the potential for arsenic remediation.

### Transport and carbohydrate uptake

The transport systems are important for microorganisms to acquire nutrients, remove harmful by-products, secrete metabolic products, and maintain the level of protons and various ions in the cytoplasm required for cell growth and division^24^. The molecules transported by the transporters produced by WSUCF1 genome are summarized in Table 3. The strain WSUCF1 has 136 genes encoding ATP-binding cassette (ABC) transporters (Supplementary Table S3), which are a superfamily of integral membrane proteins for ATP-dependent transportation of various substrates such as ions, salts, sugars, vitamins, amino acids, peptides and purine across cell membranes. Fifteen genes coding for phosphotransferase system (PTS) for specific uptake of carbohydrates are present in strain WSUCF1 genome (Supplementary Table S4). Combined with the ABC transporters and other permeases for carbohydrate transportation, WSUCF1 contains genes encoding carbohydrate transporters for the uptake of glucose, fructose, mannose, galactose, xylose, arabinose, lactose, sucrose, maltose, and cellobiose.

**Table 3:** The molecules transported by transporter systems in *Geobacillus* sp. WSUCF1.

	Molecules
Metal ions	Na^+^, K^+^, Mg^+^, Zn^+^, Cu^2+^, Ca^2+^, Mn^2+^, Fe^2+/3+^, Co^2+^, Mo^2+^, Pb^2+^, Cd^2+^, Hg^2+^
Anions	Phosphate, sulfate, nitrate, nitrite, chromate, alkanesulfonate
Other cations	Ammonium
Amino acids	Glutamine, glutamate, lysine, serine, threonine, tryptophan, aspartate, proline, arginine, methionine
Carbohydrate	Glucose, fructose, trehalose, mannitol, sucrose, xylose, galactose, maltose, ribose, maltodextrin
Other molecules	Biotin, vitamin B12, benzoate, riboflavin, glycine betaine, hydroxymethylpyrimidine, thiamine, niacin, dimethylbenzimidazole, tricarboxylate, dicarboxylate, spermidine, putrescine, glycerol-3-phosphate, urea

### EPS production

**Figure 1:**
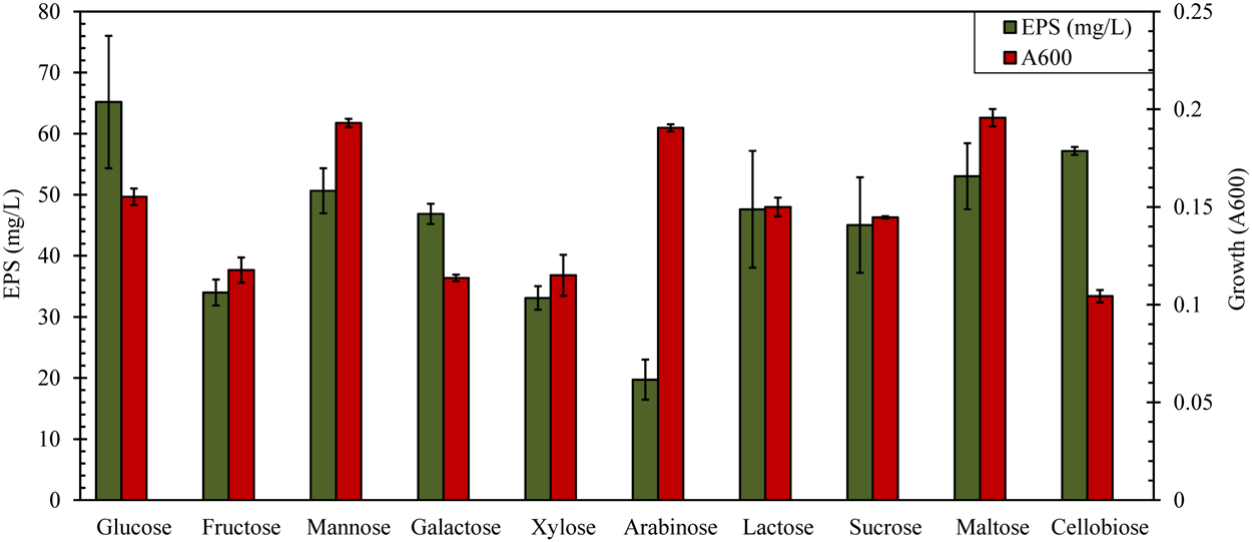
EPS production and cell growth of *Geobacillus* sp. strain WSUCF1 using different sugar carbon sources. Values are means with standard error bars.

*Geobacillus* sp. strain WSUCF1 was grown in the presence of 10 different sugars as the carbon source for EPS production. The growth profiles of strain WSUCF1 demonstrated that all these 10 sugars could be metabolized to form cell biomass, which in turn was identified by the presence of the associated transporter genes in the WSUCF1 genome. When using glucose as a carbon and energy source, the thermophilic bacterium *Geobacillus* sp. WSUCF1 produced a statistically higher amount of EPS (*p* < 0.05). Meanwhile, the amount of EPS production using cellobiose and maltose was comparable to that on glucose medium. The relatively higher cell growth was attained by using mannose, arabinose, and maltose as carbon source (Fig. 1).

### EPS biosynthesis

**Figure 2:**
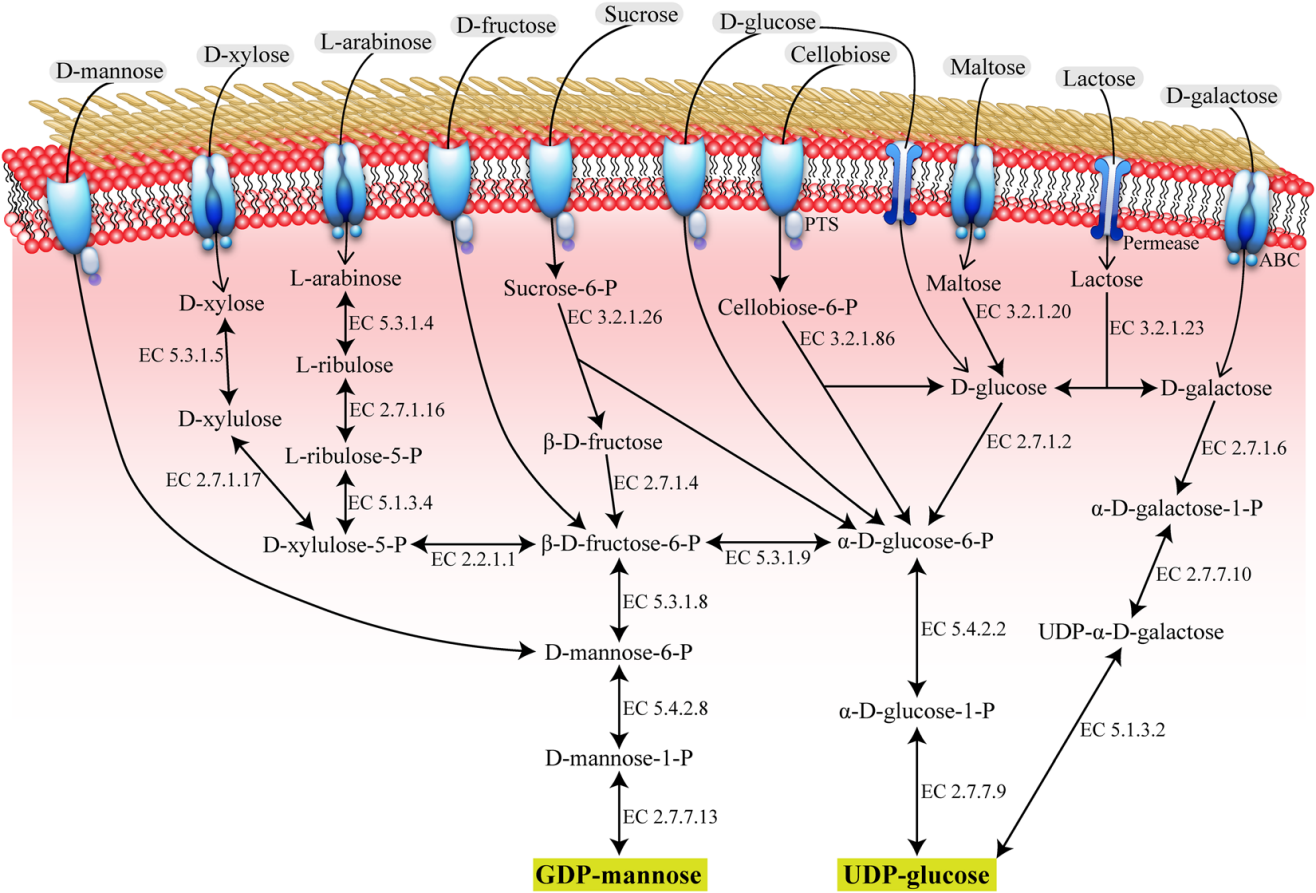
Biosynthesis of nucleotide sugars for EPS production in *Geobacillus* sp. WSUCF1 inferred from genomic sequence data. The EC numbers refer to the enzymes involved: EC 2.2.1.1, Transketolase; EC 2.7.1.2, Glucokinase; EC 2.7.1.4, Fructokinase; EC 2.7.1.6, Galactokinase; EC 2.7.1.16, Ribulokinase; EC 2.7.1.17, Xylulose kinase; EC 2.7.7.9, UTP-glucose-1-phosphate uridylyltransferase; EC 2.7.7.10, Galactose-1-phosphate uridylyltransferase; EC 2.7.7.13, Mannose-1-phosphate guanylyltransferase; EC 3.2.1.20, α-glucosidase; EC 3.2.1.23, β-galactosidase; EC 3.2.1.26, Sucrose-6-phosphate hydrolase; EC 3.2.1.86, 6-phospho-β-glucosidase; EC 5.1.3.2, UDP-glucose-4-epimerase; EC 5.1.3.4, L-ribulose-5-phosphate 4-epimerase; EC 5.3.1.4, L-arabinose isomerase; EC 5.3.1.5, Xylose isomerase; EC 5.3.1.8, Mannose-6-phosphate isomerase; EC 5.3.1.9, Glucose-6-phosphate isomerase; EC 5.4.2.2, Phosphoglucomutase; EC 5.4.2.8, Phosphomannomutase.

The annotation of *Geobacillus* sp. WSUCF1 genome demonstrated a putative biosynthesis pathway of EPSs, including synthesis of nucleoside diphosphate monosaccharides (NDP-sugars), assembly of the repeating unit as well as translocation and secretion. The initial step for EPS biosynthesis is the glycolysis pathway which is the first phase of carbohydrate catabolism. As the precursor molecules for EPS biosynthesis, NDP-sugars are derived from phosphorylated monosaccharide intermediates generated during the glycolysis process, and they are the interface between primary and secondary metabolisms^25^. The WSUCF1 strain could synthesize two types of EPSs which were glucomannan and mannan, and due to the simplicity of the EPSs synthesized by this thermophilic strain WSUCF1, only two activated precursors are required for EPS biosynthesis. The enzymes involved in UDP-glucose and GDP-mannose production, including UTP-glucose-1-phosphate uridylyltransferase (EC 2.7.7.9) and UDP-glucose-4-epimerase (EC 5.1.3.2) for UDP-glucose biosynthesis, and mannose-1-phosphate guanylyltransferase (EC 2.7.7.13) for GDP-mannose biosynthesis are all present in WSUCF1 (Supplementary Table S5). The partial sugar metabolism for nucleotide sugar biosynthesis as the preliminary model for the first stage of EPS biosynthesis mechanism of WSUCF1 is proposed in Fig. 2 using its genome information.

For the next phase of EPS biosynthesis, using the activated nucleotide sugar precursors, the EPS can be synthesized by glycosyltransferases (GTs) which can transfer the additional monosaccharides to the nascent polysaccharide chain linked on undecaprenol intermediate as membrane-associated anchor for EPS elongation. The strain WSUCF1 possessed an integrated 2-*C*-methyl-D-erythritol 4-phosphate/1-deoxy-D-xylulose 5-phosphate (MEP/DOXP) pathway for undecaprenol diphosphate synthesis (Supplementary Fig S1). Thirteen GTs (EC 2.4.1.-) were identified through dbCAN web server related with EPS biosynthesis in the WSUCF1 genome (Table 4). The sequence alignment of the GTs of strain WSUCF1 with priming GTs of *Streptococcus thermophilus*^26^, *Streptococcus agalactiae*, *Lactobacillus helveticus*^27^ and *Lactobacillus rhamnosus*^28^ was performed. The results revealed that the protein similarity of the GT encoded by WSUCF1.peg.97 with priming GT of *Streptococcus thermophilus* was 63%, and the GT coded by WSUCF1.peg.99 with priming GT of *Streptococcus agalactiae* was 83%. Therefore, it could be assumed that WSUCF1.peg.97 and WSUCF1.peg.99 encoded two priming glycosyltransferases. Among the other GTs, the GT4 family glycosyltransferases might be α-mannosyltransferase which could assemble GDP-mannose onto the nascent EPS chain and form an α-(1 → 3) glycosidic bond. The GT2 family glycosyltransferases could link UDP-glucose to the polysaccharide chain with a β-(1 → 3) or β-(1 → 4) glycoside bond. Finally, the EpsC and EpsD proteins are proposed to be involved in the EPS chain-length determination^28^.

**Table 4:** Glycosyltransferases (GTs) in *Geobacillus* sp. WSUCF1.

Feature ID	Encoded protein	Length (aa)	GT Family
WSUCF1.peg.97*	Glycosyltransferase (EC 2.4.1.-)	359	GT4
WSUCF1.peg.99*	Glycosyltransferase (EC 2.4.1.-)	418	GT4
WSUCF1.peg.256	Glycosyltransferase (EC 2.4.1.-)	360	GT4
WSUCF1.peg.670	Glycosyltransferase (EC 2.4.1.-)	330	GT2
WSUCF1.peg.703	Glycosyltransferase (EC 2.4.1.-)	285	GT2
WSUCF1.peg.1712	Glycosyltransferase (EC 2.4.1.-)	395	GT4
WSUCF1.peg.2493	Glycosyltransferase (EC 2.4.1.-)	355	GT2
WSUCF1.peg.2637	Glycosyltransferase (EC 2.4.1.-)	367	GT2
WSUCF1.peg.2919	Glycosyltransferase (EC 2.4.1.-)	320	GT2
WSUCF1.peg.3491	Glycosyltransferase (EC 2.4.1.-)	642	GT2
WSUCF1.peg.3492	Glycosyltransferase (EC 2.4.1.-)	691	GT2
WSUCF1.peg.3493	Glycosyltransferase (EC 2.4.1.-)	796	GT2
WSUCF1.peg.3913	Glycosyltransferase (EC 2.4.1.-)	340	GT2

^*^Putative priming GT.

**Figure 3:**
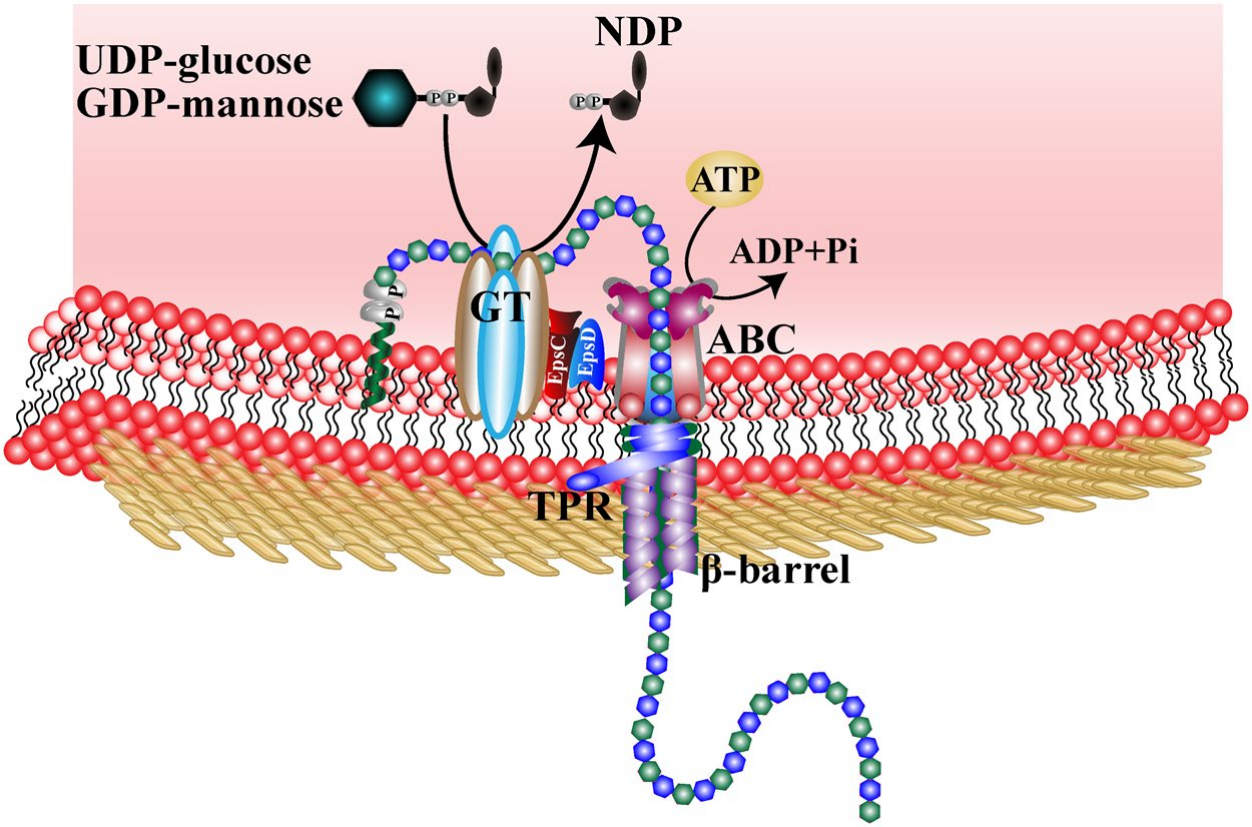
Assembly and transportation of EPS in *Geobacillus* sp. WSUCF1 inferred from genomic sequence data.

No genes encoding sucrase type enzymes, such as glucansucrase and fructansucrase, were observed in the genome of strain WSUCF1, indicating the EPSs are synthesized intracellularly like most of the EPSs from extremophilic bacteria. The secretion of bacterial EPSs generally follows one of three mechanisms: (i) Wzx/Wzy-dependent pathway; (ii) ATP-binding cassette (ABC) transporter-dependent pathway, and (iii) synthase-dependent pathway^29^. Fifteen genes were identified that encoded ABC transporters for secretion of EPS using Transporter Classification Database (TCDB) (Supplementary Table S7). The presence of these ABC transporters suggests that the EPS export of WSUCF1 follows the ABC transporter-dependent pathway. Besides, the absence of the genes encoding Wzx flippase, Wzy polymerase, and polysaccharide synthase also indicates that WSUCF1 secretes EPS through ABC transporter-dependent mechanism. Using the genome information of WSUCF1, a preliminary model for the export process of EPS is depicted in Fig. 3.

### Regulatory system of EPS biosynthesis

**Figure 4:**
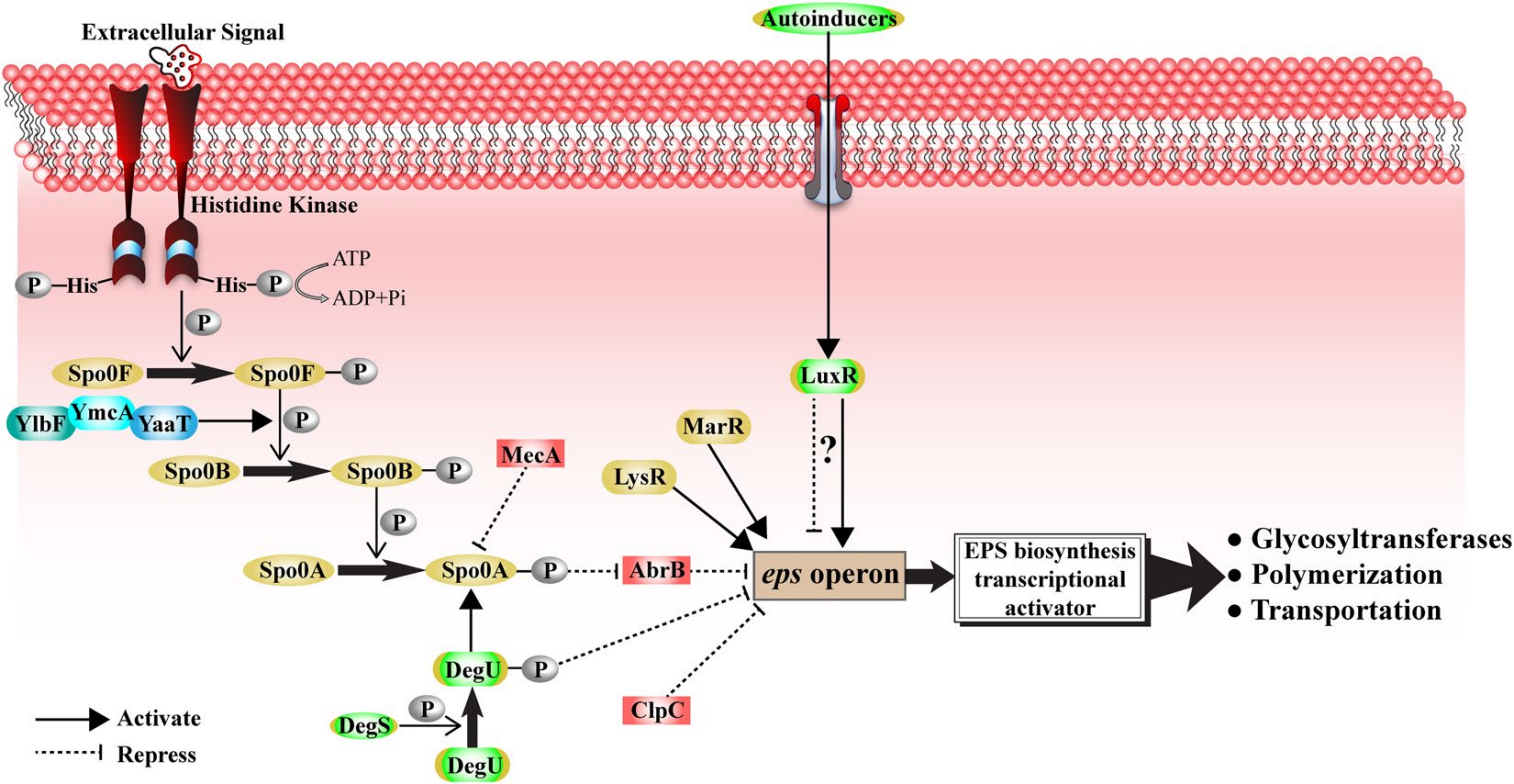
Predicted regulatory network for EPS biosynthesis in *Geobacillus* sp. WSUCF1 inferred from genomic sequence data.

The biosynthesis of macromolecules is an energetically expensive process. Therefore, the transcription of the genes for the synthesis of thermophilic EPS should be tightly regulated by a sophisticated genetic network which permits each cell to respond to the extreme environment effectively. Based on the genome annotation results, the genes coding for regulatory systems are present in the WSUCF1 genome. The predicted regulatory network correlated with EPS biosynthesis is shown in Fig. 4.

EPS biosynthesis is able to be induced after recognition of extracellular signals by membrane-located sensors^30^. For the thermophilic strain WSUCF1, based on the hypothetical regulatory pathway of EPS production, the presence of genes encoding histidine kinase in WSUCF1 genome indicates that the input of phosphoryl moiety for phosphorelay may be transferred by a group of histidine kinases due to their functions. The histidine kinase could also identify the extracellular signal molecules which are synthesized by the control of quorum sensing^31^. It is proposed that the function of response regulator protein Spo0F is to receive a phosphate signal from the histidine kinases, and then transfer the phosphoryl group from phosphorylated Spo0F (Spo0F-P) to phosphotransferase protein Spo0B and finally to the response regulator Spo0A to generate phosphorylated Spo0A (Spo0A-P)^32^. In addition, the genes coding for YmcA, YlbF and YaaT proteins were all detected in the WSUCF1 genome. YmcA and YlbF could interact with YaaT to form a tripartite complex, which may influence the phosphorelay of Spo0A by affecting the phosphotransfer between Spo0F and Spo0B^33^. Finally, the Spo0A-P may also be relevant to the expression of the gene encoding AbrB protein, which is a direct repressor for the transcription of EPS biosynthetic genes^32^.

Besides the master regulation by Spo0A for EPS biosynthesis, some other regulators can also mediate the EPS production in thermophilic strain WSUCF1. MecA was speculated to bind the Spo0A-P and inhibit its transcriptional activity^34^, thus downregulating the expression of EPS biosynthetic genes. The ATP-dependent protease ClpC may play a negative role in the regulation of EPS gene expression since the inactivation of gene encoding ClpC leads to overexpression of EPS genes^34^. On the other hand, MarR family regulatory protein was assigned to be an activator for the expression of EPS biosynthesis genes^35,36^. LysR type transcription factors were also considered to be involved in the activation of EPS synthetic genes^37,38^, which could serve as a future avenue of exploration.

Several regulators were found in WSUCF1 genome, but their functions for EPS biosynthesis are still unknown. DegU is a response regulator phosphorylated by its cognate histidine kinase DegS^39^, and both of their genes are present in the WSUCF1 genome. The phosphorylated DegU (DegU-P) increases the level of Spo0A-P. However, a high level of DegU-P blocked biofilm formation by the transcriptional inhibition of EPS gene promoters^39^. Thus, DegU may function as a dual-purpose regulator for EPS biosynthesis. LuxR is a receptor for *N*-acylhomoserine lactones (AHLs) which are quorum sensing regulatory signals for gene expression^40^. Although the genes for LuxR family protein were detected in the genome of WSUCF1, no genes encoding LuxI family protein could be observed to constitute an integrated LuxI/LuxR quorum sensing system.

## Discussion

The *Geobacillus* sp. strain WSUCF1 is a cellulose-degrading bacterium (CDB) which produces a complete repertoire of highly thermostable lignocellulolytic enzymes^41^. For lignocellulose deconstruction, both the cellulase and xylanase from WSUCF1 demonstrated outstanding thermostability and could be promising for the development of thermophilic consolidated bioprocessing to convert recalcitrant lignocellulosic woody biomass into biofuels and value-added bioproducts^18,19,22,42^. In our current investigation, strain WSUCF1 was also able to produce significant amounts of EPS, which was comparable with other reported *Geobacillus* species EPS producers^14,17,43^, with developing applications in biomedical and biomaterial industries. The whole-genome annotation of *Geobacillus* sp. strain WSUCF1 was performed in this study to model the genetic and metabolic network for thermophilic EPS biosynthesis. To our knowledge, no genome of any *Geobacillus* sp. strain has been completely annotated for its EPS biosynthesis pathways.

It is now widely accepted that extremophilic microorganisms will be a valuable resource for exploitation in novel biotechnological processes, such as synthesis of EPSs with uncommon physicochemical properties^7^. The thermophilic EPS from *G*. *thermodenitrificans* B3–72 could be used in a potential therapeutic strategy for equilibrating the immune response in viral diseases^13^. *Geobacillus* sp. strain TS3–9 produced an EPS which demonstrated strong antioxidant activity against reactive oxygen species^17^. The EPS from *Geobacillus* sp. 1A60 possessed heavy metal binding capability which could be appealing as cast hybrid films for filtration^14^. The biosynthetic and regulatory mechanism elucidated in this study for the EPS produced by *Geobacillus* sp. strain WSUCF1 will merit further research for biomedical and biomaterial application of these bioactive thermophilic EPSs.

Genome annotation can be an effective tool to assign the essential genes involved in EPS biosynthesis and thus serve as the starting point for further metabolic reconstruction for EPS overproduction by extremophiles. For instance, the genomic analysis for a halophilic bacterium *Halomonas smyrnensis* AAD6^T^ and its EPS production has been performed^44^, and then the gene coding for fructose-specific PTS was knocked out to obtain an engineered strain with redirected metabolism which demonstrated higher efficiency of EPS production and substrate conversion compared with the wild-type strain^45^. Meanwhile, boric acid was also shown to be a strong stimulator for the EPS production by halophilic strain *Halomonas smyrnensis* AAD6^T^. It was speculated that the boric acid mediated accumulation of quorum sensing signal furanosyl borate diester autoinducer (AI-2) could stimulate the GT expression involved in EPS biosynthesis and then lead to higher EPS production^46^. The synthesis of impurities which are difficult to remove by physicochemical methods during EPS purification could also be blocked by deleting related genes to obtain a higher yield and purity of EPS^47^.

The availability of genome annotation information also makes thermophilic bacterium *Geobacillus* sp. WSUCF1 a potential candidate as a microbial factory for large-scale EPS production. The identification of genes coding for EPS production within the genome of strain WSUCF1 could provide engineering strategies for both enhanced EPS production and rational design space for tailor-made EPS. In the NDP-sugar biosynthesis phase, the improvement of NDP-sugar precursor level could be exploited through the augmentation of critical enzymes in the central metabolism for supplying nucleotide sugar^48^. The mutant with improved EPS production demonstrated that the specific activities of phosphoglucomutase (5.4.2.2), UDP-glucose pyrophosphorylase (2.7.7.9) and UDP-galactose-4-epimerase (5.1.3.2) were higher than those of the wild-type strain^49–51^, indicating potential targets for improvement of EPS production in strain WSUCF1 through homologous overexpression. The expression level of enzymes associated with GDP-mannose synthesis may also be important since multiple interventions during NDP-sugar synthesis are more likely to be efficient to promote EPS production^52,53^. As the second stage for EPS biosynthesis, GTs are responsible for the assembly of activated NDP-sugars to the polysaccharide chain. The increased EPS production could be achieved by the overexpression of the gene coding for priming GT^54^. Moreover, the disruption of genes encoding one of the non-priming GTs within the EPS biosynthesis system was proved to be able to modify EPS monomer composition, since the addition of a certain type of monosaccharide unit onto EPS chain could be blocked^27^. Finally, it was considered that the overexpression of the genes coding for the proteins involved in EPS transport system (e.g. ABC transporter) might be able to dictate the EPS chain length^55,56^.

*Geobacillus* sp. strain WSUCF1 was able to utilize 10 types of sugars as carbon and energy source for both biomass formation and EPS production. The results demonstrated that higher cell growth does not correspond to higher EPS production, such as in the case where arabinose was used. This phenomenon may relate to the complexity of the metabolic pathway for NDP-sugar biosynthesis. The low EPS producing level for the carbon source with a simple NDP-sugar synthesis pathway (e.g. fructose) may due to the low activity of its transporter. The optimum carbon source for EPS production by *Geobacillus* sp. WSUCF1 was glucose, which may be attributed to the strain WSUCF1 possessing both the genes coding for PTS and permease for glucose transportation. Besides the potential engineering strategies mentioned above, the overexpression of these glucose-specific transporters could be a design space for a higher rate of substrate uptake to improve the fermentation performance of EPS production. Moreover, the wide spectrum of carbon source utilization makes strain WSUCF1 feasible to metabolize mixed sugar substrates efficiently. Combined with the strong lignocellulolytic capability, there is potential for strain WSUCF1 to utilize complex lignocellulosic substrates for EPS production through thermophilic consolidated bioprocessing.

The three general mechanisms of EPS production and secretion: Wzx/Wzy-dependent pathway, ABC transporter-dependent pathway and synthase-dependent pathway, have already been reviewed^29,57–59^. However, these three mechanisms are mainly for the EPS biosynthesis by Gram-negative bacteria. The general mechanisms of EPS biosynthesis in Gram-positive bacteria have not been systematically reviewed. Due to the absence of the outer membrane, the transmembrane mechanisms of EPSs of Gram-positive bacteria may be different from those of Gram-negative bacteria. Gram-positive strain *Geobacillus* sp. WSUCF1 uses ABC transporters to export the EPS across the plasma membrane, followed by translocation across the periplasm through tetratricopeptide repeat (TPR) protein and β-barrel protein. In the ABC-dependent pathway, the EPS is completely assembled at the cytoplasmic site of the plasma membrane through the sequential addition of monosaccharide units to the polymer chain before release by ABC transporter^57,58^. TPR proteins can be the scaffold proteins for the assembly of the EPS secretion complex, and the association of TPR with GGDEF domain protein is also required for the export of biopolymer^24^. Although several genes encoding lipoproteins are present in the WSUCF1 genome, these lipoproteins cannot be identified as the outer membrane polysaccharide export (OPX) family of proteins in a Gram-positive bacterium. Meanwhile, no genes encoding polysaccharide co-polymerase (PCP) were detected in the WSUCF1 genome. Both PCP and OPX are responsible for EPS export across periplasm and outer membrane in the ABC-dependent pathway in Gram-negative bacteria. The group of TPR and β-barrel proteins are considered as the export mechanism of synthase-dependent pathway for EPS secretion in Gram-negative bacteria. However, based on the genome annotation information of Gram-positive bacterium WSUCF1, TPR and β-barrel proteins are putatively combined with ABC transporter as an integrated system for EPS export across the plasma membrane and periplasm. This special type of ABC-dependent pathway for EPS transport was also found in a thermophilic Gram-positive bacterium *Brevibacillus thermoruber* strain 423 through genome analysis^24^.

The regulation of EPS biosynthesis is a multi-gene involved process. The YmcA-YlbF-YaaT ternary complex is required to increase the level of phosphorylated Spo0A above the threshold needed for induction of EPS biosynthesis^33^. The Spo0B is considered as rate limiting in the process of phosphorelay, and Spo0B can be a control point for phosphorylated Spo0A formation. The down-regulation of genes encoding YmcA and YlbF is also considered to be involved in a negative feedback which is dependent on phosphorylated Spo0A. For the overproduction of EPS, an appropriate Spo0A-P level is also critical in order to avoid the negative feedback in transcription of genes of YmcA and YlbF and also the proceeding of sporulation at excess Spo0A-P level^33^. The EPS production may benefit from the absence of the genes encoding proteins with inhibitory effects on the phosphorelay process of Spo0A. The phosphorelay can be a central signal integration which provides the correct level of Spo0A-P to induce EPS biosynthesis in order to protect against the extreme environment. During EPS production, the formation of Spo0A-P should not be interfered with, to avoid any increased rate of transcription of AbrB gene^32^. The increased level of DegU-P may be helpful to minimize the energy used by cells for EPS production^39^. After a threshold level of DegU-P, the EPS biosynthetic genes would not be over-transcribed due to its transcriptional inhibition effect to the EPS gene promoters. DegU may also be required for growth at high temperatures, and DegU synthesis can be regulated by itself but whether the effect of its self-regulation can be positive or negative is still controversial^60^.

Both AbrB and SinR inhibit the transcription of the operons required for EPS biosynthesis^61^. Due to the loss of SinR in the WSUCF1 genome, AbrB is not capable of working synergistically with SinR for the negative control of EPS biosynthesis, and it can be speculated that the absence of SinR may be beneficial for the overproduction of extremophilic EPS with specific structure and properties against harsh conditions. MecA is able to dampen EPS production and prevent inappropriate expression of EPS biosynthetic pathway, and this mechanism may buffer the energy-intensive process and maximize fitness and survival^34^.

The orphan LuxR proteins are present in the genome of Gram-positive bacterium WSUCF1. Although LuxS synthase for AI-2 was found in WSUCF1 genome, the sensor kinase LuxQ as a detector for AI-2 is absent. According to the genome completeness estimates using CheckM tool, the genome of strain WSUCF1 is a nearly complete genome (completeness >90%) with low level of contamination (<5%), suggesting that the fraction of missed genes in contig gaps is minimal^62,63^. Thus, it can be speculated that AI-2 could not influence the EPS production through quorum sensing circuit due to the lack of the sensory transportation channel to LuxR from AI-2 signal molecule in WSUCF1. Another pathway for the activation of LuxR is the AHL autoinducer produced by LuxI family protein. The AHL-dependent quorum sensing is generally responsible for the intraspecies bacterial communication^64^. WSUCF1 lacks the gene encoding autoinducer synthase LuxI, indicating that the signal could be from other species. The LuxI/LuxR system is usually considered as a model quorum sensing system in Gram-negative bacteria, and the LuxR sequences in Gram-positive bacteria may be acquired from Gram-negative bacteria through horizontal gene transfer (HGT)^65^. The presence of LuxR strengthens the existence of quorum sensing in strain WSUCF1, but due to the uncertain regulatory effect of LuxR to EPS biosynthetic genes, the regulation of EPS production through AHL autoinducer still requires further exploration to confirm the regulatory pathways.

In summary, the genome of thermophilic strain *Geobacillus* sp. WSUCF1 was annotated to explain its EPS synthesis, export and regulatory signals. The elucidation of strain WSUCF1 genome makes this strain a model thermophilic bacterium for EPS production among the *Geobacillus* species strains. Meanwhile, the putative EPS biosynthetic and regulatory pathways built in this study could provide opportunities to develop metabolic engineering strategies for the strain WSUCF1 to attain enhanced EPS production, which is also significant for further feasibility studies on industrial-scale applications of the EPSs produced by other *Geobacillus* strains. The specific features disclosed in this study also reveal the utility of *Geobacillus* sp. strain WSUCF1 as a multifunctional industrial strain, and possibilities for exploiting the sophisticated adaptation systems of thermophiles.

## Methods

### Bacterium strain and sequence

The draft genome of thermophilic *Geobacillus* sp. strain WSUCF1 which was sequenced and assembled in our previous work^41^ was downloaded from the NCBI database. Sequencing data for strain WSUCF1 are available online as BioProject PRJNA192273, NCBI taxonomy ID 886559. The Whole Genome Shotgun project of strain WSUCF1 was deposited at DDBJ/EMBL/GenBank under the accession number ATCO00000000.

### Gene prediction and annotation

The genome of strain WSUCF1 was uploaded to the web annotation service Pathosystems Resource Integration Center (PATRIC; https://www.patricbrc.org/)^66^ as well as Rapid Annotations using Subsystems Technology (RAST; http://rast.nmpdr.org/rast.cgi)^67^ for automated annotation followed by manual scan. The PATRIC gene features were chosen as a basis for annotation. The output from PATRIC was assessed and validated by comparing to that from RAST Server, and the analysis of genomic and metabolic pathways was performed using both PATRIC and RAST. These data sources were combined to assert product description for predicted proteins. The gene features of essential biosystems were also manually verified using BLASTp (https://blast.ncbi.nlm.nih.gov/Blast.cgi) against National Center for Biotechnology Information (NCBI) non-redundant database and re-annotated as necessary. The amino acid sequences encoded by annotated genes were compared with all proteins from complete microbial genomes, and the alignment length over 90% of its own length and over 60% match identity were selected as candidates. The best BLAST hit with highest alignment length percentage and match identity was assigned as the annotation of predicted gene^68^. The functions of essential enzymes were obtained from Kyoto Encyclopedia of Genes and Genomes (KEGG; http://www.genome.jp/kegg/). Then the metabolic pathways related to EPS biosynthesis were reconstructed based on the functional annotation. The sequence alignment of the glycosyltransferases of strain WSUCF1 with priming GTs was performed using BLASTp (https://blast.ncbi.nlm.nih.gov/Blast.cgi). The CRISPR/Cas systems in strain WSUCF1 were identified with CRISPR finder (http://crispr.i2bc.paris-saclay.fr/Server/). The glycosyltransferases for EPS biosynthesis were further identified using dbCAN2 meta server (http://cys.bios.niu.edu/dbCAN2/index.php)^69^. The genes coding for ABC transporter involved in EPS export were verified using the similarity searches against the Transporter Classification Database (TCDB) (www.tcdb.org), and annotations of best-matching hits for ABC transporter genes were transferred with an E value cutoff of 1e-9^70,71^. The completeness and contamination of the genome of strain WSUCF1 was assessed using CheckM tool^72^.

### EPS production

The *Geobacillus* sp. WSUCF1 strain was grown in a liquid medium containing (g/L) glucose, 6.0; yeast extract, 1.0; and NaCl, 3.0. To test the EPS production with other sugar carbon sources, the glucose in the medium was replaced by 6.0 g/L mannose, galactose, arabinose, fructose, xylose, lactose, maltose, sucrose, and cellobiose, respectively. The pH was adjusted to 7.0 with 6 M NaOH. The strain was inoculated into 500-mL Erlenmeyer flasks containing 200 mL of the medium supplemented with each of the ten different sugars mentioned above, and incubated at 60 °C, 180 rpm for 24 h. The cell growth was evaluated by measuring optical density at 600 nm (OD_600_). Then the cells in broth were removed by centrifugation at 6000 rpm for 15 min at 4 °C. The supernatant was treated with an equal volume of chilled absolute ethanol added dropwise under stirring in an ice bath, and then incubated at -20 °C overnight. The pellets were recovered by centrifugation at 8000 rpm for 40 min at 4 °C. Afterwards, the precipitated EPS was washed two times with absolute ethanol and dissolved in deionized water, dialyzed against deionized water for 72 h at 4 °C, and then lyophilized. This sample was tested for carbohydrate content through phenol-sulfuric acid method using glucose as standard^73^. All the experiments were carried out in triplicates and appropriate controls, e.g. culture- and substrate-free, were run with each experiment. The statistical analysis of the experimental data was determined by one-way analysis of variance (ANOVA) using Microsoft Excel. Significance of difference was determined as *p* < 0.05.

## Supplementary information

Supplementary Information

## Data Availability

The datasets generated during and/or analyzed during the current study are available from the corresponding author on reasonable request.
