# Supplementary material for: Genome analysis of a thermophilic exopolysaccharide-producing bacterium - Geobacillus sp. WSUCF1
Source: Sci Rep. 2019 Feb 7;9:1608. doi: 10.1038/s41598-018-36983-z (PMC6367360; doi:10.1038/s41598-018-36983-z)
Supplement: Supplementary file 1 — Supplementary Information [file 41598_2018_36983_MOESM1_ESM.docx]

**Supplementary Information for**

**Genome analysis of a thermophilic exopolysaccharide-producing bacterium - *Geobacillus* sp. WSUCF1**

Jia Wang^a,e^, Kian Mau Goh^b^, David R. Salem^a,c,d,^*, and Rajesh K. Sani^a,d,e,^*^^[[1]](#footnote-1)^^

^a^ Department of Chemical and Biological Engineering, South Dakota School of Mines and Technology, Rapid City, SD 57701, USA

^b^ Faculty of Science, Universiti Teknologi Malaysia, Skudai, Johor 81300, Malaysia

^c^ Department of Materials and Metallurgical Engineering, South Dakota School of Mines and Technology, Rapid City, SD 57701, USA

^d^ Composite and Nanocomposite Advanced Manufacturing – Biomaterials Center (CNAM-Bio Center), Rapid City, SD 57701, USA

^e^ BuG ReMeDEE Consortium, South Dakota School of Mines and Technology, Rapid City, SD 57701, USA

**Methods**

**Comparative genome analysis**

To compare the genome of WSUCF1 with the genomes of other *Geobacillus* spp., the following unannotated genomes were downloaded from NCBI database and analyzed by RAST server based on SEED subsystems for functional annotations^1^: *G. thermoleovorans* CCB_US3_UF5 (NCBI accession number NC_016593), *G. thermoleovorans* FJAT-2391 (NCBI accession number NZ_CP017071), *G. thermoleovorans* N7 (NCBI accession number NZ_MDCP00000000), *G. thermoleovorans* B23 (NCBI accession number NZ_BATY00000000), and *G. zalihae* NBRC 101842 (NCBI accession number NZ_BCPV00000000).

**Results and Discussion**

**Regulators in quorum sensing**

Quorum sensing is defined as the regulation of a group of multicellular activities in bacteria by cell density. The biological process such as EPS biosynthesis can be influenced by bacterial population density via cell-cell communication, and Gram-positive bacteria use peptides as extracellular cell-to-cell signaling molecules ^2,3^. The oligopeptide-mediated quorum sensing system of strain WSUCF1 includes genes encoding oligopeptide autoinducer precursor, histidine kinase receptor, and cognate cytoplasmic response regulator (transcriptional regulator).

ABC exporter proteins are responsible to secrete the autoinducers for quorum sensing. Meanwhile, the signaling peptides could be internalized by the oligopeptide permease Opp, which is ATP-binding cassette transporter composed of five periplasmic oligopeptide-binding proteins including OppA, OppB, OppC, OppD and OppF. The regulator CodY positively controls the production of OppABCDF ^4^.

The TetR transcriptional repressing family is abundant in bacteria exposed to complex and changing environments, and this type of regulator is important for bacteria to adapt environmental fluctuations ^5,6^. TetR family proteins can regulate their own synthesis in feedback control in order to keep the level of transcriptional repressors in an optimal range ^7^.

SpaK is an orphan sensor histidine kinase which senses the subtilin for quorum sensing control of subtilin biosynthesis. However, WSUCF1 does not have the gene encoding its corresponding response regulator SpaR. The other genes for biosynthesis of subtilin are all absent in the WSUCF1 genome.

ComQ is for the modification and secretion of active ComX peptide pheromone from pre-ComX. Sensor histidine kinase ComP can detect the extracellular signal molecule (ComX peptide pheromone). However, the gene coding for cognate response regulator ComA is absent in WSUCF1 genome. Therefore, the transformation chain of phosphorylation from ComA to DegU was not completed in the WSUCF1 genome. Hfq protein controls interactions between regulatory RNAs (sRNAs) and specific messenger RNA (mRNA) targets, to mediate the stability of the target transcripts. Hfq is also required for quorum sensing repression ^8^.

**Natural competence and sporulation**

Natural competence and sporulation are two physiological processes controlled by quorum sensing. Natural competence is the capability of a bacterium to take up extracellular DNA and incorporate the newly acquired DNA in its own genome, leading to a permanent change in its phenotype ^9^. Sporulation occurs under extreme stress and lack of nutrition conditions. The autoinducer peptides are considered to regulate natural competence and sporulation in a quorum sensing dependent manner ^10^.

Thermophilic strain WSUCF1 has evolved a highly developed sporulation system for the formation of endospore (Supplementary Table S10). Generally, the sporulation system of a thermophilic strain can be more developed than mesophilic EPS-producing bacteria ^11^, indicating that sporulation is likely to be more important for extremophiles. The endospore formed through sporulation can be highly resistant, such that it can retain adaptive function for extremophilic conditions.

WSUCF1 has an integrated system for natural competence. However, the regulatory system of competence of WSUCF1 may differ from that of mesophilic bacteria, which means thermophilic bacteria use a different regulatory machinery for competence ^10^. The horizontal gene transfer through competence may be the driving force in the evolution of thermophilic species, and acquisition of exogenous DNA leads to genetic plasticity which may improve the strains’ survival under extreme conditions ^12^. Furthermore, the imported DNA can be applied as templates for DNA repair ^13^. On the other hand, the extreme conditions in a thermophilic environment may be an inducing condition for competence of thermophiles ^14^. Study of the competence of WSUCF1 can be significant for the development of natural DNA transformation protocols to make progress in the genetic engineering of *Geobacillus*.

**Defense system**

*Geobacillus* sp. strain WSUCF1 possesses the genes encoding both functional multi antimicrobial extrusion (MATE) family multidrug resistance (MDR) efflux pump and MDR ABC transporters (Supplementary Table S12). MDR is defined as acquired non-susceptibility to at least one agent in three or more antimicrobial categories ^15^. The multidrug resistance efflux pumps coded by pathogenic bacterial confer resistance to antibiotics in clinics ^16^. Most of the thermophiles are considered as non-pathogenic strains, and it is more likely these efflux pumps encoded by thermophiles have physiological roles. One possible function can be to confer to the bacteria resistance to natural substances in their ecological niche. The efflux pumps can transport a range of structurally different chemicals. The expression of certain types of efflux pumps usually requires an inducer when the bacterium is growing in its natural niche ^16^. The multidrug ABC transporters can be effective in transporting cytotoxic compounds to the extracellular environment. Among the multidrug resistance genes, no gene encoding lactamase was detected in the WSUCF1 genome. The strain WSUCF1 shows the presence of the genes for penicillin binding proteins, fosmidomycin and daunorubicin resistance proteins (Supplementary Table S12) which may be obtained through horizontal gene transfer. In addition, strain WSUCF1 exhibits genes related to lantibiotic transport and biosynthesis. Lantibiotic can be applied as antibiotics against other microorganisms such as food-borne pathogens ^11^. Lantibiotic biosynthetic genes are commonly distributed in *Geobacillus* species ^17^.

In some prokaryotes, clustered regularly interspaced short palindromic repeats (CRISPR) and CRISPR-associated sequence (Cas) proteins can be an antiviral defense system against the bacteriophage infection. After the integration of new spacers derived from phage genomic sequences, the strain with CRISPR can be resistant to the infection by phages due to the CRISPR-mediated phage resistance. The transcripts of these spacers can be applied as guide RNAs to destruct the cognate foreign DNA or RNA using Cas proteins ^18^. The CRISPR system leads to changes in the genome of bacteria and provides selective pressure ^19,20^. Therefore, the CRISPR can provide immunological memory for bacteria by the heritable persistence of the spacers ^18^. In the genome of strain WSUCF1, five CRISPR loci were identified by CRISPR Finder, but only one CRISPR locus was identified to associate with genes encoding Cas proteins (Supplementary Figure S2). For the future industrial application of WSUCF1, the CRISPR system may provide a potential solution for the phage infection problem by artificially integrating spacers derived from certain phages to induce immunity to the phages. The genomic distribution of CRISPR defense systems can be different between thermophiles and mesophiles due to the degree of invasion, and CRISPR sequences are considered to be significantly more prevalent in thermophiles than in mesophiles ^21,22^.

The genes coding for the restriction-modification (R-M) system were detected in the WSUCF1 genome. The R-M system is another defense system protecting bacteria from alien DNA. R-M and CRISPR-Cas systems may comprise the host-defense system in WSUCF1 and work synergistically to improve the phage resistance of the bacteria ^9^. The genes encoding both type I and III R-M systems are present in the WSUCF1 genome. The CRISPR-Cas and R-M systems of WSUCF1 may influence the genetic accessibility and contribute to phage resistance in the microbiome of the compost where the WSUCF1 strain was isolated.

**Features of biotechnological interest**

The identification of genes related to the valuable enzymes could contribute to the development of industrially significant processes using this thermophilic bacterium. Due to the rampant deployment of conventional antibiotics, the emergence of antibiotic-resistance is quite common in more and more pathogenic bacteria. Therefore, the use of traditional antibiotics cannot be an effective long-term solution for treating bacterial infections, and the application of novel antibacterial reagents has become necessary. The quorum quenching enzymes have been considered to be one of the alternative antibiotics to control the infection of pathogenic bacteria by interfering with the quorum sensing systems and thus suppressing the expression of the virulence factors involved in triggering their pathogenic phenotype ^23^. Besides antibiotic applications, biofilm formation controlled by quorum sensing can be interrupted by quorum quenching enzymes, in order to mitigate biofouling on membranes caused by biofilm growth. The thermostable quorum quenching enzymes is also suitable to be applied in seawater cooling tower systems with challenging environmental factors, to inhibit sulfate-reducing bacteria which promote biofouling and biocorrosion ^24^. The thermostable quorum quenching lactonase from *Geobacillus* species has a broad AHL substrate spectrum and can be active in a wide temperature range up to 70°C ^25^. Therefore, the AHL lactonase from thermophilic bacterium WSUCF1 may be an excellent candidate for the control and prevention of pathogens and biofouling effect.

Cas6 is an endoribonuclease which can be present in a wide range of prokaryotes with their CRISPR-Cas system. The Cas6 homolog has been used as biotechnology tool for purification of tagged RNA transcripts, post-transcriptional regulation of protein expression and targeted genome editing ^26^. The Cas6 protein from thermophilic strain *Geobacillus* sp. WSUCF1 could be potential for targeted cleavage requiring elevated temperature conditions. The thermostable Cas protein may enhance the utility of CRISPR-Cas technology at higher temperature, and enable feasible genome editing in thermophiles using biotechnology which was limited in mesophilic microorganisms ^27^.

BpsA is a type III polyketide synthase which catalyzes the long-chain fatty acyl-coenzyme A (CoA) thioesters as starter substrate and malonyl-CoA as extender substrate to generate triketide pyrone. The methyltransferase BspB can then methylate the triketide pyrone synthesized by BspA to generate triketide pyrone methyl ether ^28^. Discovery of thermostable type III polyketide synthase is appealing to provide a robust biosynthetic tool for industrial application due to its broad substrate specificity ^29^.

**Comparative *Geobacillus* genome analysis**

Strain WSUCF1 was identified by 16S rRNA gene sequence analysis as *Geobacillus* genus ^30^. The comparison of 16S rRNA sequence has been applied as a conventional sequence-based method for classifying a newly discovered bacterium to higher taxa. Currently, due to the improvement and cost reduction in DNA sequencing techniques, the whole genome sequences can be used in microbial taxonomy to provide more accurate circumscription. Average Nucleotide Identity (ANI) method has been considered as a superior substitute for microbial species demarcation and phylogeny ^31^. *Geobacillus* sp. WSUCF1 has been assigned as *G. thermoleovorans* via ANI from the most recent report ^32^. Meanwhile, in the same report, the closest relative of strain WSUCF1 is *G. zalihae* NBRC 101842 from the phylogenetic network ^32^. Therefore, the functional annotation of WSUCF1 was compared with other four sequenced *G. thermoleovorans* strains and *G. zalihae* NBRC 101842.

The genome sizes of the six selected strains were in the range of 3.35 to 3.60 Mb. Supplementary Figure S3 shows the analysis of genome classification of the six strains using RAST genome annotation database. Generally, the number of genes in each functional group is comparable among all six strains. The functional groups with significantly higher numbers of genes in these six strains are those associated with carbohydrates, amino acids and derivatives, protein metabolism, and cofactors, vitamins, prosthetic groups, pigments. This indicates the above four functional groups may be closely related to the adaptation of *Geobacillus thermoleovorans* to thermophilic environments, considering the fluctuation of carbon source and other nutrients. These six *Geobacillus* species strains demonstrate a similar number of stress response sequences, because they are all thermophiles. WSUCF1 demonstrates the highest number of genes in the carbohydrate group, which may be associates with its outstanding lignocellulolytic and EPS biosynthetic capabilities.

Table S1. Stress tolerance

| Feature ID | Encoded protein |
| --- | --- |
| ***Heat adaptation*** | |
| WSUCF1.peg.1463 | Spermidine/putrescine import ABC transporter ATP-binding protein PotA (TC 3.A.1.11.1) |
| WSUCF1.peg.1464 | Spermidine/putrescine import ABC transporter permease protein PotB (TC 3.A.1.11.1) |
| WSUCF1.peg.1465 | Spermidine/putrescine import ABC transporter permease protein PotC (TC 3.A.1.11.1) |
| WSUCF1.peg.1466 WSUCF1.peg.3807 | Spermidine/putrescine import ABC transporter substrate-binding protein PotD (TC 3.A.1.11.1) |
| WSUCF1.peg.1655 | DNA gyrase subunit A (EC 5.99.1.3) |
| WSUCF1.peg.1656 WSUCF1.peg.1657 | DNA gyrase subunit B (EC 5.99.1.3) |
| WSUCF1.peg.1695 | S-adenosylmethionine decarboxylase proenzyme (EC 4.1.1.50), prokaryotic class 1B |
| WSUCF1.peg.1835 | S-adenosylmethionine decarboxylase proenzyme (EC 4.1.1.50), prokaryotic class 1A |
| WSUCF1.peg.2106 | DNA-binding protein HU |
| WSUCF1.peg.2188 WSUCF1.peg.3371 | Spermidine synthase (EC 2.5.1.16) |
| WSUCF1.peg.3372 | Agmatinase (EC 3.5.3.11) |
| WSUCF1.peg.3402 WSUCF1.peg.4041 | Arginine decarboxylase (EC 4.1.1.19) |
| ***Heat response*** | |
| WSUCF1.peg.1 | tmRNA-binding protein SmpB |
| WSUCF1.peg.213 | Translation elongation factor LepA |
| WSUCF1.peg.214 | Hypothetical radical SAM family enzyme in heat shock gene cluster |
| WSUCF1.peg.215 | Heat-inducible transcription repressor HrcA |
| WSUCF1.peg.216 | Heat shock protein GrpE |
| WSUCF1.peg.217 | Chaperone protein DnaK |
| WSUCF1.peg.218 | Chaperone protein DnaJ |
| WSUCF1.peg.219 | Ribosomal protein L11 methyltransferase |
| WSUCF1.peg.221 | tRNA t(6)A37 methylthiotransferase (EC 2.8.4.5) |
| WSUCF1.peg.935 WSUCF1.peg.936 | tRNA t(6)A37 methylthiotransferase (EC 2.8.4.3) |
| WSUCF1.peg.486 | Ribosome-associated heat shock protein implicated in the recycling of the 50S subunit (S4 paralog) |
| WSUCF1.peg.502 | Chaperonin (heat shock protein 33) |
| WSUCF1.peg.783 | ATP-dependent Clp protease, ATP-binding subunit ClpX |
| WSUCF1.peg.1639 | ATP-dependent Clp protease, ATP-binding subunit ClpE |
| WSUCF1.peg.3359 | ATP-dependent Clp protease proteolytic subunit (EC 3.4.21.92) |
| WSUCF1.peg.796 | FIG009886: phosphoesterase |
| WSUCF1.peg.797 | Ribonuclease PH (EC 2.7.7.56) |
| WSUCF1.peg.973 | Transcriptional regulator CtsR |
| WSUCF1.peg.1306 WSUCF1.peg.1307 | ATP-dependent HSL protease ATP-binding subunit HslU |
| WSUCF1.peg.1308 | ATP-dependent protease subunit HslV (EC 3.4.25.2) |
| WSUCF1.peg.1545 | Heat shock protein 60 family chaperone GroEL |
| WSUCF1.peg.1546 | Heat shock protein 60 family co-chaperone GroES |
| WSUCF1.peg.2005 | Hypothetical radical SAM family enzyme, NOT coproporphyrinogen III oxidase, oxygen-independent |
| WSUCF1.peg.2624 | Heat shock protein |
| WSUCF1.peg.2867 | RNA polymerase heat shock sigma factor SigI |
| WSUCF1.peg.3394 | Small heat shock protein |
| WSUCF1.peg.3440 | Stress response HSP-like protein |
| WSUCF1.peg.4014 | tRNA (adenine37-N(6))-methyltransferase TrmN6 (EC 2.1.1.223) |
| WSUCF1.peg.4015 | DNA replication initiation control protein YabA |
| ***Oxidative stress*** | |
| WSUCF1.peg.377 WSUCF1.peg.2202 WSUCF1.peg.2374 | NAD-dependent protein deacetylase of SIR2 family |
| WSUCF1.peg.567 WSUCF1.peg.1365 WSUCF1.peg.1845 WSUCF1.peg.2497 WSUCF1.peg.3369 WSUCF1.peg.3815 | Thioredoxin reductase (EC 1.8.1.9) |
| WSUCF1.peg.1036 WSUCF1.peg.1865 WSUCF1.peg.2417 | Thioredoxin |
| WSUCF1.peg.1258 | Superoxide dismutase [Fe] (EC 1.15.1.1) |
| WSUCF1.peg.1505 | Nitric oxide-responding transcriptional regulator NnrA (Crp/Fnr family) |
| WSUCF1.peg.1693 | NADPH-dependent glyceraldehyde-3-phosphate dehydrogenase (EC 1.2.1.13) |
| WSUCF1.peg.2037 | Ferric uptake regulation protein FUR |
| WSUCF1.peg.2190 | Nitrite-sensitive transcriptional repressor NsrR |
| WSUCF1.peg.2191 | Flavohermoglobin/Nitric oxide dioxygenase (EC 1.14.12.17) |
| WSUCF1.peg.2875 | Peroxide stress regulator PerR, FUR family |
| WSUCF1.peg.3028 | Cold shock protein of CSP family |
| WSUCF1.peg.3079 | Nicotinamidase (EC 3.5.1.19) |
| WSUCF1.peg.3104 | Superoxide dismutase [Cu-Zn] precursor (EC 1.15.1.1) |
| WSUCF1.peg.3353 | NAD-dependent glyceraldehyde-3-phosphate dehydrogenase (EC 1.2.1.12) |
| WSUCF1.peg.3574 | Zinc uptake regulation protein ZUR |
| WSUCF1.peg.3587 | Superoxide dismutase [Mn] (EC 1.15.1.1) |
| ***Acid resistance*** | |
| WSUCF1.peg.2091 | Urease gamma subunit (EC 3.5.1.5) |
| WSUCF1.peg.2092 | Urease beta subunit (EC 3.5.1.5) |
| WSUCF1.peg.2093 | Urease alpha subunit (EC 3.5.1.5) |
| WSUCF1.peg.2094 | Urease accessory protein UreE |
| WSUCF1.peg.2095 | Urease accessory protein UreF |
| WSUCF1.peg.2096 | Urease accessory protein UreG |
| WSUCF1.peg.2097 WSUCF1.peg.2098 | Urease accessory protein UreD |
| ***Carbon starvation*** |  |
| WSUCF1.peg.1919 WSUCF1.peg.3980 | Carbon starvation protein A |
| WSUCF1.peg.3488 | Carbon storage regulator |
| ***Osmotic stress*** | |
| WSUCF1.peg.223 | Sodium-dependent phosphate transporter |
| WSUCF1.peg.1381 | KtrAB potassium uptake system, integral membrane component KtrB |
| WSUCF1.peg.1677 | Potassium efflux system KefA protein |
| WSUCF1.peg.1944 | KtrCD potassium uptake system, integral membrane component KtrD |
| WSUCF1.peg.2878 | Potassium channel protein (PCP) |
| WSUCF1.peg.3091 | Potassium voltage-gated channel subfamily KQT |
| WSUCF1.peg.3737 | KtrCD potassium uptake system, peripheral membrane component KtrC |
| WSUCF1.peg.881 | Ca^2+^/H^+^ antiporter |
| WSUCF1.peg.2183 | Na^+^/H^+^ antiporter |
| WSUCF1.peg.2732 WSUCF1.peg.2733 | NhaC, Na^+^/H^+^ antiporter |
| WSUCF1.peg.2817 | Putative sodium-glucose/galactose cotransporter |
| WSUCF1.peg.3033 | Glycerol uptake facilitator protein |
| WSUCF1.peg.4132 | Glycine betaine transporter OpuD |
| ***DNA repair*** | |
| WSUCF1.peg.310 | Uracil-DNA glycosylase, family 1 (EC 3.2.2.27) |
| WSUCF1.peg.449 WSUCF1.peg.3470 | ATP-dependent DNA helicase UvrD/PcrA (EC 3.6.4.12) |
| WSUCF1.peg.875 | DNA-3-methyladenine glycosylase II (EC 3.2.2.21) |
| WSUCF1.peg.972 | Nucleotide excision repair protein, with UvrB/UvrC motif |
| WSUCF1.peg.1691 | Formamidopyrimidine-DNA glycosylase (EC 3.2.2.23) |
| WSUCF1.peg.2901 | A/G-specific adenine glycosylase (EC 3.2.2.-) |
| WSUCF1.peg.250 | Endonuclease IV (EC 3.1.21.2) |
| WSUCF1.peg.2039 | Endonuclease Q, cleaves 5’ to damaged DNA bases |
| WSUCF1.peg.2612 | Endonuclease III (EC 4.2.99.18) |
| WSUCF1.peg.2734 | Endonuclease I |
| WSUCF1.peg.70 | 2',3'-cyclic-nucleotide 2'-phosphodiesterase (EC 3.1.4.16)/3'-nucleotidase (EC 3.1.3.6) |
| WSUCF1.peg.618 | Glycerophosphoryl diester phosphodiesterase (EC 3.1.4.46) |
| WSUCF1.peg.2406 | Alkaline phosphodiesterase I (EC 3.1.4.1)/Nucleotide pyrophosphatase (EC 3.6.1.9) |
| WSUCF1.peg.3594 | Diguanylate cyclase/phosphodiesterase (GGDEF & EAL domains) with PAS/PAC sensor(s) |
| WSUCF1.peg.3653 | Glycerophosphoryl diester phosphodiesterase, periplasmic (EC 3.1.4.46) |
| WSUCF1.peg.448 | DNA ligase (NAD(+)) (EC 6.5.1.2) |
| WSUCF1.peg.940 | DNA mismatch repair protein MutS |
| WSUCF1.peg.941 | DNA mismatch repair protein MutL |
| WSUCF1.peg.927 WSUCF1.peg.4023 | RecA protein |
| WSUCF1.peg.2666 | Recombination protein RecR |
| WSUCF1.peg.1426 | DNA polymerase III subunits gamma and tau (EC 2.7.7.7) |
| WSUCF1.peg.1660 WSUCF1.peg.1661 | DNA polymerase III beta subunit (EC 2.7.7.7) |
| WSUCF1.peg.1689 | DNA polymerase I (EC 2.7.7.7) |
| WSUCF1.peg.2074 WSUCF1.peg.3701 | DNA polymerase beta domain protein region |
| WSUCF1.peg.2426 WSUCF1.peg.2427 | DNA polymerase X family |
| WSUCF1.peg.2538 WSUCF1.peg.2539 | DNA polymerase III delta subunit (EC 2.7.7.7) |
| WSUCF1.peg.3330 | DNA polymerase III polC-type (EC 2.7.7.7) |
| WSUCF1.peg.3531 | DNA polymerase III alpha subunit (EC 2.7.7.7) |
| WSUCF1.peg.4017 WSUCF1.peg.4018 | DNA polymerase III delta prime subunit (EC 2.7.7.7) |
| WSUCF1.peg.199 WSUCF1.peg.3167 WSUCF1.peg.3168 WSUCF1.peg.3169 | Single-stranded-DNA-specific exonuclease RecJ (EC 3.1.-.-) |
| WSUCF1.peg.547 WSUCF1.peg.749 | Single-stranded DNA-binding protein |
| WSUCF1.peg.555 | ATP-dependent DNA helicase RecS (RecQ family) |
| WSUCF1.peg.744 | Replicative DNA helicase (DnaB) (Ec 3.6.4.12) |
| WSUCF1.peg.1191 WSUCF1.peg.1192 | RecD-like DNA helicase YrrC |
| WSUCF1.peg.2955 | ATP-dependent DNA helicase RecG (EC 3.6.4.12) |
| WSUCF1.peg.2973 | Helicase PriA |
| WSUCF1.peg.3179 | Holliday junction DNA helicase RuvB |
| WSUCF1.peg.3180 | Holliday junction DNA helicase RuvA |
| ***Other stress tolerance genes*** | |
| WSUCF1.peg.11 WSUCF1.peg.3219 | Anaerobic regulatory protein Fnr |
| WSUCF1.peg.105 WSUCF1.peg.3764 | Cell envelope-associated transcriptional attenuator LytR-CpsA-Psr, subfamily F2 |
| WSUCF1.peg.276 | Hemoglobin-like protein HbO |
| WSUCF1.peg.304 WSUCF1.peg.1529 WSUCF1.peg.1704 | Serine phosphatase RsbU, regulator of sigma subunit |
| WSUCF1.peg.306 WSUCF1.peg.307 WSUCF1.peg.1530 | Serine-protein kinase RsbW (EC 2.7.11.1) |
| WSUCF1.peg.308 | Anti-sigma B factor antagonist RsbV |
| WSUCF1.peg.892 | Sulfate permease, Pit-type |
| WSUCF1.peg.1247 | Sensor histidine kinase ResE (EC 2.7.13.3) |
| WSUCF1.peg.1248 | DNA-binding response regulator ResD |
| WSUCF1.peg.1506 WSUCF1.peg.1507 | Nitric-oxide reductase (EC 1.7.99.7), quinol-dependent |
| WSUCF1.peg.2030 | Anti-sigma F factor antagonist |
| WSUCF1.peg.2997 | Ribosome LSU-associated GTP-binding protein HflX |
| WSUCF1.peg.3080 | Nicotinate phosphoribosyltransferase (EC 6.3.4.21) |
| WSUCF1.peg.3098 WSUCF1.peg.3126 | General stress protein |
| WSUCF1.peg.3165 | Guanosine-3’,5’-bis(diphosphate) 3’-pyrophosphohydrolase (EC 3.1.7.2)/GTP pyrophosphokinase (EC 2.7.6.5), (p)ppGpp synthetase II |
| WSUCF1.peg.3547 | Universal stress protein family |

Table S2. Genes with industrial potential

| Feature ID | Encoded protein | |
| --- | --- | --- |
| ***Hydrolase*** | | |
| WSUCF1.peg.653 WSUCF1.peg.1784 | alpha-amylase (EC 3.2.1.1) | |
| WSUCF1.peg.1013 WSUCF1.peg.2672 | Arabinan endo-1,5-alpha-L-arabinosidase (EC 3.2.1.99) | |
| WSUCF1.peg.1014 WSUCF1.peg.2631 | Alpha-galactosidase (EC 3.2.1.22) | |
| WSUCF1.peg.1779 | Neopullulanase (EC 3.2.1.135) | |
| WSUCF1.peg.2058 | Beta-glucosidase (EC 3.2.1.21) | |
| WSUCF1.peg.2379 | Alpha-mannosidase (EC 3.2.1.24) | |
| WSUCF1.peg.2433 WSUCF1.peg.2728 WSUCF1.peg.2822 WSUCF1.peg.3973 | 6-phospho-beta-glucosidase (EC 3.2.1.86) | |
| WSUCF1.peg.2633 WSUCF1.peg.3387 | Beta-galactosidase (EC 3.2.1.23) | |
| WSUCF1.peg.2690 WSUCF1.peg.2696 WSUCF1.peg.2707 WSUCF1.peg.2708 | Xylan 1,4-beta-xylosidase (EC 3.2.1.37) | |
| WSUCF1.peg.2691 WSUCF1.peg.2704 | Endo-1,4-beta-xylanase (EC 3.2.1.8) | |
| WSUCF1.peg.2695 | Xylan alpha-1,2-glucuronosidase (EC 3.2.1.131) | |
| WSUCF1.peg.3301 WSUCF1.peg.3957 | Endoglucanase M | |
| WSUCF1.peg.3949 | Pullulanase (EC 3.2.1.41) | |
| WSUCF1.peg.3977 | Alpha-glucosidase (EC 3.2.1.20) | |
| ***Others*** |  | |
| WSUCF1.peg.136 | Acetaldehyde dehydrogenase, acetylating, (EC 1.2.1.10) in gene cluster for degradation of phenols, cresols, catechol |  |
| WSUCF1.peg.192 WSUCF1.peg.2834 | Arsenical resistance operon repressor |  |
| WSUCF1.peg.193 WSUCF1.peg.2833 | Arsenical-resistance protein ACR3 |  |
| WSUCF1.peg.194 WSUCF1.peg.1861 WSUCF1.peg.2832 | Arsenate reductase (EC 1.20.4.1) |  |
| WSUCF1.peg.427 WSUCF1.peg.428 WSUCF1.peg.3109 | Arsenic efflux pump protein |  |
| WSUCF1.peg.1030 WSUCF1.peg.1722 | Transcriptional repressor, ArsR family |  |
| WSUCF1.peg.342 | Thiol:disulfide oxidoreductase related to ResA |  |
| WSUCF1.peg.350 | Alkylpyrone O-methyltransferase BpsB |  |
| WSUCF1.peg.351 WSUCF1.peg.352 WSUCF1.peg.353 | Type III polyketide synthase BpsA |  |
| WSUCF1.peg.594 | O-methyltransferase involved in polyketide biosynthesis |  |
| WSUCF1.peg.712 | Regulator of polyketide synthase expression |  |
| WSUCF1.peg.406 WSUCF1.peg.1021 WSUCF1.peg.1152 WSUCF1.peg.2269 WSUCF1.peg.2420 WSUCF1.peg.2934 WSUCF1.peg.2935 | Enoyl-CoA hydratase (EC 4.2.1.17) |  |
| WSUCF1.peg.817 | lipase |  |
| WSUCF1.peg.1158 WSUCF1.peg.1335 WSUCF1.peg.1362 WSUCF1.peg.3522 | Alcohol dehydrogenase (EC 1.1.1.1) |  |
| WSUCF1.peg.2651 | NADPH-dependent butanol dehydrogenase |  |
| WSUCF1.peg.2689 | Chitin deacetylase |  |
| WSUCF1.peg.3955 | Quorum-quenching lactonase YtnP |  |

Table S3. ATP-binding cassette (ABC) transporters in *Geobacillus* sp. WSUCF1

| Feature ID | Encoded protein |
| --- | --- |
| WSUCF1.peg.10 | Molybdenum ABC transporter permease protein ModB |
| WSUCF1.peg.1462 | Molybdenum ABC transporter ATP-binding protein ModC |
| WSUCF1.peg.3292 | Molybdenum ABC transporter, substrate-binding protein ModA |
| WSUCF1.peg.29 WSUCF1.peg.2758 WSUCF1.peg.2759 | Methionine ABC transporter ATP-binding protein |
| WSUCF1.peg.2757 | Methionine ABC transporter permease protein |
| WSUCF1.peg.2756 | Methionine ABC transporter substrate-binding protein |
| WSUCF1.peg.174 | Branched-chain amino acid ABC transporter, amino acid-binding protein (TC 3.A.1.4.1) |
| WSUCF1.peg.254 | Zinc ABC transporter, ATP-binding protein ZnuC |
| WSUCF1.peg.255 WSUCF1.peg.3573 | Zinc ABC transporter, permease protein ZnuB |
| WSUCF1.peg.368 WSUCF1.peg.1374 WSUCF1.peg.1392 WSUCF1.peg.1471 WSUCF1.peg.1523 WSUCF1.peg.1565 WSUCF1.peg.1646 WSUCF1.peg.1802 WSUCF1.peg.2007 WSUCF1.peg.2567 WSUCF1.peg.2884 WSUCF1.peg.3628 WSUCF1.peg.3703 WSUCF1.peg.3709 WSUCF1.peg.3791 | ABC transporter (3.A.1.132.1), for secretion of exopolysaccharide |
| WSUCF1.peg.389 | Branched-chain amino acid ABC transporter, amino acid-binding protein (TC 3.A.1.4.1) |
| WSUCF1.peg.401 WSUCF1.peg.1555 WSUCF1.peg.1556 WSUCF1.peg.3245 | ABC transporter, ATP-binding protein |
| WSUCF1.peg.676 WSUCF1.peg.677 | Cell-division-associated, ABC-transporter-like signaling protein FtsX |
| WSUCF1.peg.678 | Cell-division-associated, ABC-transporter-like signaling protein FtsE |
| WSUCF1.peg.718 | ABC transporter amino acid-binding protein |
| WSUCF1.peg.719 | Glutamine transport system permease protein glnP |
| WSUCF1.peg.720 | Glutamine transport ATP-binding protein glnQ |
| WSUCF1.peg.826 WSUCF1.peg.827 WSUCF1.peg.828 | Glutamine ABC transporter, permease protein GlnP |
| WSUCF1.peg.829 | Glutamine ABC transporter, substrate-binding protein GlnH |
| WSUCF1.peg.830 | Glutamine ABC transporter, ATP-binding protein GlnQ |
| WSUCF1.peg.977 | Maltodextrin ABC transporter, permease protein MdxG |
| WSUCF1.peg.978 | Maltodextrin ABC transporter, permease protein MdxF |
| WSUCF1.peg.980 | Maltodextrin ABC transporter, substrate-binding protein MdxE |
| WSUCF1.peg.1238 | Vitamin B12 ABC transporter, ATP-binding protein BtuD |
| WSUCF1.peg.1239 | Vitamin B12 ABC transporter, permease protein BtuC |
| WSUCF1.peg.1240 | Vitamin B12 ABC transporter, substrate-binding protein BtuF |
| WSUCF1.peg.1375 WSUCF1.peg.1376 | Export ABC transporter permease protein |
| WSUCF1.peg.1463 | Spermidine/putrescine import ABC transporter ATP-binding protein PotA (TC 3.A.1.11.1) |
| WSUCF1.peg.1464 | Spermidine/putrescine import ABC transporter permease protein PotB (TC 3.A.1.11.1) |
| WSUCF1.peg.1465 | Spermidine/putrescine import ABC transporter permease protein PotC (TC 3.A.1.11.1) |
| WSUCF1.peg.1466 WSUCF1.peg.3807 | Spermidine/putrescine import ABC transporter substrate-binding protein PotD (TC 3.A.1.11.1) |
| WSUCF1.peg.1533 WSUCF1.peg.1534 WSUCF1.peg.3009 WSUCF1.peg.3010 | β-Glucan export ABC transporter permease protein (3.A.1.108.1) |
| WSUCF1.peg.1645 WSUCF1.peg.2568 WSUCF1.peg.2880 WSUCF1.peg.2881 WSUCF1.peg.3244 WSUCF1.peg.3631 WSUCF1.peg.3993 | ABC transporter permease protein |
| WSUCF1.peg.1780 WSUCF1.peg.1781 | Maltodextrin ABC transporter, substrate-binding protein MdxE |
| WSUCF1.peg.1782 | Maltodextrin ABC transporter, permease protein MdxF |
| WSUCF1.peg.1783 | Maltodextrin ABC transporter, permease protein MdxG |
| WSUCF1.peg.1788 | ABC-type antimicrobial peptide transport system, ATPase component |
| WSUCF1.peg.1789 | ABC-type antimicrobial peptide transport system, permease component |
| WSUCF1.peg.1893 | Urea carboxylase-related ABC transporter, permease protein |
| WSUCF1.peg.1895 | Urea carboxylase-related ABC transporter, ATPase protein |
| WSUCF1.peg.2086 | Urea ABC transporter, substrate binding protein UrtA |
| WSUCF1.peg.2087 | Urea ABC transporter, permease protein UrtB |
| WSUCF1.peg.2088 | Urea ABC transporter, permease protein UrtC |
| WSUCF1.peg.2089 | Urea ABC transporter, ATPase protein UrtD |
| WSUCF1.peg.2090 WSUCF1.peg.2161 | Urea ABC transporter, ATPase protein UrtE |
| WSUCF1.peg.2291 | Predicted β-glucoside-regulated ABC transport system, sugar binding component |
| WSUCF1.peg.2295 WSUCF1.peg.2848 | ABC transporter, substrate-binding protein (cluster 2, ribose/xylose/arabinose/galactose) |
| WSUCF1.peg.2413 | Alkanesulfonate ABC transporter ATP-binding protein SsuB |
| WSUCF1.peg.2414 | Alkanesulfonate ABC transporter permease protein SsuC |
| WSUCF1.peg.2488 | ABC-type transporter ATP-binding protein ecsA, partial |
| WSUCF1.peg.2685 | Xylose ABC transporter, substrate-binding component |
| WSUCF1.peg.2687 | Xylose ABC transporter, ATP-binding component |
| WSUCF1.peg.2692 | Maltose ABC transporter |
| WSUCF1.peg.2693 WSUCF1.peg.2694 WSUCF1.peg.4183 | Sugar ABC transporter permease |
| WSUCF1.peg.2846 WSUCF1.peg.3468 | Ribose ABC transport system, permease protein RbsC (TC 3.A.1.2.1) |
| WSUCF1.peg.2847 WSUCF1.peg.3466 WSUCF1.peg.3467 | Ribose ABC transport system, ATP-binding protein RbsA (TC 3.A.1.2.1) |
| WSUCF1.peg.3465 | Ribose ABC transport system, high affinity permease RbsD (TC 3.A.1.2.1) |
| WSUCF1.peg.3469 | Ribose ABC transport system, periplasmic ribose-binding protein RbsB (TC 3.A.1.2.1) |
| WSUCF1.peg.2891 | Dipeptide-binding ABC transporter, periplasmic substrate-binding component (TC 3.A.1.5.2) |
| WSUCF1.peg.2948 WSUCF1.peg.2949 | Glycerol-3-phosphate ABC transporter, ATP-binding protein UgpC (TC 3.A.1.1.3) |
| WSUCF1.peg.2950 | Glycerol-3-phosphate ABC transporter, permease protein UgpA (TC 3.A.1.1.3) |
| WSUCF1.peg.2951 | Glycerol-3-phosphate ABC transporter, permease protein UgpE (TC 3.A.1.1.3) |
| WSUCF1.peg.2952 | Glycerol-3-phosphate ABC tranporter, substrate-binding protein UgpB |
| WSUCF1.peg.3003 | ABC transport protein, sugar-binding component YneA |
| WSUCF1.peg.3142 | Hydroxymethylpyrimidine ABC transporter, transmembrane component |
| WSUCF1.peg.3143 | Hydroxymethylpyrimidine ABC transporter, ATPase component |
| WSUCF1.peg.3144 | ABC transporter substrate-binding protein |
| WSUCF1.peg.3243 | BMP family ABC transporter substrate-binding protein |
| WSUCF1.peg.3411 WSUCF1.peg.3412 | Zinc ABC transporter, substrate-binding protein ZnuA |
| WSUCF1.peg.3580 | Phosphate ABC transporter, periplasmic phosphate-binding protein PstS (TC 3.A.1.7.1) |
| WSUCF1.peg.3881 | ABC transporter periplasmic binding protein yphF |
| WSUCF1.peg.3899 WSUCF1.peg.3900 | ABC-type Fe^3+^ -siderophore transport system, periplasmic iron-binding component |
| WSUCF1.peg.3901 WSUCF1.peg.3902 | ABC-type Fe^3+^-siderophore transport system, permease component |
| WSUCF1.peg.3903 | ABC-type Fe^3+^-siderophore transport system, permease 2 component |
| WSUCF1.peg.3904 WSUCF1.peg.3905 | ABC-type Fe^3+^-siderophore transport system, ATP-binding protein |
| WSUCF1.peg.3997 | Nitrate ABC transporter, permease protein |
| WSUCF1.peg.4033 | Purine nucleoside ABC transporter, permease protein 2 |
| WSUCF1.peg.4034 WSUCF1.peg.4035 | Purine nucleoside ABC transporter, permease protein 1 |
| WSUCF1.peg.4036 | Purine nucleoside ABC transporter, ATP-binding protein |
| WSUCF1.peg.4123 | Purine nucleoside ABC transporter, substrate-binding protein |
| WSUCF1.peg.4144 | Dipeptide-binding ABC transporter, periplasmic substrate-binding component (TC 3.A.1.5.2) |

Table S4. Carbohydrate transport

| Feature ID | Encoded protein | |
| --- | --- | --- |
| ***Phosphotransferase system (PTS)*** | |  |
| WSUCF1.peg.527 | PTS system, fructose-specific IIABC component |  |
| WSUCF1.peg.708 WSUCF1.peg.2180 | PTS system, sucrose-specific IIBC component |  |
| WSUCF1.peg.710 | PTS system, glucose-specific IIA component |  |
| WSUCF1.peg.1243 | PTS system, N-acetylglucosamine-specific IIBC component |  |
| WSUCF1.peg.1617 | PTS system, glucose-specific IIABC component |  |
| WSUCF1.peg.2435 WSUCF1.peg.4048 | PTS system, mannose-specific IIABC component |  |
| WSUCF1.peg.2725 | PTS system, cellobiose-specific IIB component |  |
| WSUCF1.peg.2726 WSUCF1.peg.3017 | PTS system, cellobiose-specific IIA component |  |
| WSUCF1.peg.2727 | PTS system, cellobiose-specific IIC component |  |
| WSUCF1.peg.4051 WSUCF1.peg.4052 WSUCF1.peg.4147 | PTS system, mannitol-specific IIBC component |  |
| ***Permease*** | | |
| WSUCF1.peg.2289 WSUCF1.peg.2290 | Glucose transport system permease protein | |
| WSUCF1.peg.2686 | Xylooligosaccharide transporter system permease protein | |
| WSUCF1.peg.3385 | Lactose transport system permease protein LacF | |
| WSUCF1.peg.3386 | Lactose transport system permease protein LacG | |

Table S5. EPS biosynthesis – NDP sugars

| Feature ID | Encoded protein | Substrate | Product |
| --- | --- | --- | --- |
| WSUCF1.peg.706 WSUCF1.peg.707 | Sucrose-6-phosphate hydrolase (EC 3.2.1.26) | Sucrose-6-phosphate | D-glucose-6-phosphate and D-fructose |
| WSUCF1.peg.731 | Mannose-6-phosphate isomerase (EC 5.3.1.8) | β-D-fructose-6-phosphate | D-mannose-6-phosphate |
| WSUCF1.peg.1015 | L-arabinose isomerase (EC 5.3.1.4) | L-arabinose | L-ribulose |
| WSUCF1.peg.1016 WSUCF1.peg.1017 | Ribulokinase (EC 2.7.1.16) | L-ribulose | L-ribulose-5-phosphate |
| WSUCF1.peg.1018 | L-ribulose-5-phosphate 4-epimerase (EC 5.1.3.4) | L-ribulose-5-phosphate | D-xylulose-5-phosphate |
| WSUCF1.peg.1039  WSUCF1.peg.1710 | Phosphoglucomutase (EC 5.4.2.2) | α-D-glucose-6-phosphate | α-D-glucose-1-phosphate |
| WSUCF1.peg.1709 | Phosphomannomutase (EC 5.4.2.8) | D-mannose-6-phosphate | D-mannose-1-phosphate |
| WSUCF1.peg.1713 | Mannose-1-phosphate guanylyltransferase (EC 2.7.7.13) | D-mannose-1-phosphate | GDP-mannose |
| WSUCF1.peg.1943WSUCF1.peg.3758 | UTP-glucose-1-phosphate uridylyltransferase (EC 2.7.7.9) | α-D-glucose-1-phosphate | UDP-glucose |
| WSUCF1.peg.2277 | Fructokinase (EC 2.7.1.4) | D-fructose | β-D-fructose-6-phosphate |
| WSUCF1.peg.2633 WSUCF1.peg.3387 | β-galactosidase (EC 3.2.1.23) | Lactose | D-galactose and D-glucose |
| WSUCF1.peg.2709 | Xylose isomerase (EC 5.3.1.5) | D-xylose | D-xylulose |
| WSUCF1.peg.2710 | Xylulose kinase (EC 2.7.1.17) | D-xylulose | D-xylulose-5-phosphate |
| WSUCF1.peg.2433 WSUCF1.peg.2728 WSUCF1.peg.2822 WSUCF1.peg.3973 | 6-phospho-beta-glucosidase (EC 3.2.1.86) | Cellobiose-6-phosphate | D-glucose and D-glucose-6-phosphate |
| WSUCF1.peg.2453 | Glucokinase (EC 2.7.1.2) | D-glucose | α-D-glucose-6-phosphate |
| WSUCF1.peg.2655WSUCF1.peg.3093 | Glucose-6-phosphate isomerase (EC 5.3.1.9) | β-D-fructose-6-phosphate | α-D-glucose-6-phosphate |
| WSUCF1.peg.3006 | Transketolase (EC 2.2.1.1) | D-xylulose-5-phosphate | β-D-fructose-6-phosphate |
| WSUCF1.peg.3389 WSUCF1.peg.3390 | Galactokinase (EC 2.7.1.6) | D-galactose | α-D-galactose-1-phosphate |
| WSUCF1.peg.3391 | UDP-glucose-4-epimerase (EC 5.1.3.2) | UDP-α-D-galactose | UDP-glucose |
| WSUCF1.peg.3392 | Galactose-1-phosphate uridylyltransferase (EC 2.7.7.10) | α-D-galactose-1-phosphate | UDP-α-D-galactose |
| WSUCF1.peg.3977 | Alpha-glucosidase (EC 3.2.1.20) | Maltose | D-glucose |

Table S6. EPS biosynthesis – 2-*C*-methyl-D-erythritol 4-phosphate/1-deoxy-D-xylulose 5-phosphate (MEP/DOXP) pathway

| Feature ID | Encoded protein | Substrate | Product |
| --- | --- | --- | --- |
| WSUCF1.peg.247 | 4-hydroxy-3-3methylbut-2-enyl diphosphate reductase (EC 1.17.7.4) | 1-hydroxy-2-methyl-2-butenyl 4-diphosphate | Isopentenyl diphosphate |
| WSUCF1.peg.471 | 4-diphosphocytidyl-2-C-methyl-D-erythritol kinase (EC 2.7.1.148) | 4-(cytidine 5’-diphospho)-2-C-methyl-D-erythritol | 2-phospho-4-(cytidine 5’-diphospho)-2-C-methyl-D-erythritol |
| WSUCF1.peg.626 | 1-deoxy-D-xylulose 5-phosphate synthase (EC 2.2.1.7) | D-glyceraldehyde 3-phosphate and pyruvate | 1-deoxy-D-xylulose 5-phosphate |
| WSUCF1.peg.628 | (2E,6E)-farnesyl diphosphate synthase (EC 2.5.1.10) | Isopentenyl diphosphate | (2E,6E)-Farnesyl diphosphate |
| WSUCF1.peg.964 | 2-C-methyl-D-erythritol 2,4-cyclodiphosphate synthase (EC 4.6.1.12) | 2-phospho-4-(Cytidine 5’-diphospho)-2-C-methyl-D-erythritol | 2-C-methyl-D-erythritol 2,4-cyclodiphosphate |
| WSUCF1.peg.965 | 2-C-methyl-D-erythritol 4-phosphate cytidylyltransferase (EC 2.7.7.60) | 2-C-methyl-D-erythritol 4-phosphate | 4-(cytidine 5’-diphospho)-2-C-methyl-D-erythritol |
| WSUCF1.peg.1387 | Undecaprenyl-diphosphatase (EC 3.6.1.27) | di-trans, octa-cis-undecaprenyl diphosphate | di-trans, octa-cis-undecaprenyl phosphate |
| WSUCF1.peg.3333 | 1-deoxy-D-xylulose 5-phosphate reductoisomerase (EC 1.1.1.267) | 1-deoxy-D-xylulose 5-phosphate | 2-C-methyl-D-erythritol 4-phosphate |
| WSUCF1.peg.3336 | Undecaprenyl diphosphate synthase (EC 2.5.1.31) | (2E,6E)-Farnesyl diphosphate | di-trans, octa-cis-undecaprenyl diphosphate |
| WSUCF1.peg.3577 | 1-hydroxy-2-methyl-2-(E)-butenyl 4-diphosphate synthase (EC 1.17.7.1) | 2-C-methyl-D-erythritol 2,4-cyclodiphosphate | 1-hydroxy-2-methyl-2-butenyl 4-diphosphate |

Table S7. EPS biosynthesis – transport

| Feature ID | Encoded protein |
| --- | --- |
| WSUCF1.peg.368 WSUCF1.peg.1374 WSUCF1.peg.1392 WSUCF1.peg.1471 WSUCF1.peg.1523 WSUCF1.peg.1565 WSUCF1.peg.1646 WSUCF1.peg.1802 WSUCF1.peg.2007 WSUCF1.peg.2567 WSUCF1.peg.2884 WSUCF1.peg.3628 WSUCF1.peg.3703 WSUCF1.peg.3709 WSUCF1.peg.3791 | ABC transporter (3.A.1.132.1), for secretion of exopolysaccharide |
| WSUCF1.peg.640 WSUCF1.peg.641 WSUCF1.peg.734 | GGDEF domain protein |
| WSUCF1.peg.786 | TPR repeat protein |
| WSUCF1.peg.1190WSUCF1.peg.2130 WSUCF1.peg.2498 WSUCF1.peg.3073 WSUCF1.peg.3075 | Tetratricopeptide repeat (TPR) family protein |
| WSUCF1.peg.3373 | β-barrel protein YwiB |

Table S8. EPS biosynthesis – regulation

| Feature ID | Encoded protein |
| --- | --- |
| WSUCF1.peg.33 WSUCF1.peg.3473 | Transcriptional regulator protein DegU |
| WSUCF1.peg.112 WSUCF1.peg.565 | Negative regulator of genetic competence MecA |
| WSUCF1.peg.195 WSUCF1.peg.2334 WSUCF1.peg.2862 WSUCF1.peg.3622 WSUCF1.peg.3623 | Transcriptional regulator, MarR family |
| WSUCF1.peg.411 WSUCF1.peg.531 WSUCF1.peg.1177 WSUCF1.peg.1372 WSUCF1.peg.1397 WSUCF1.peg.1708 WSUCF1.peg.1790 WSUCF1.peg.2626 | Two-component transcriptional response regulator, LuxR family |
| WSUCF1.peg.482 | Stage V sporulation protein T, AbrB family transcriptional regulator (SpoVT) |
| WSUCF1.peg.620 | Stage 0 sporulation two-component response regulator (Spo0A) |
| WSUCF1.peg.937 | YmcA protein |
| WSUCF1.peg.951 | Sporulation initiation phosphotransferase (Spo0F) |
| WSUCF1.peg.969 WSUCF1.peg.970 | ATP-dependent Clp protease, ATP-binding subunit ClpC |
| WSUCF1.peg.2255 | ComK regulator YlbF |
| WSUCF1.peg.2744 WSUCF1.peg.3437 | Transcriptional regulator, LysR family |
| WSUCF1.peg.3196 | Sporulation initiation phosphotransferase B (Spo0B) |
| WSUCF1.peg.3472 | Sensor protein DegS |
| WSUCF1.peg.3766 | Tyrosine-protein kinase EpsD |
| WSUCF1.peg.3767 | Tyrosine-protein kinase transmembrane modulator EpsC |
| WSUCF1.peg.4016 | Stage 0 sporulation protein YaaT |

Table S9. Regulators in quorum sensing

| Feature ID | Encoded protein |
| --- | --- |
| WSUCF1.peg.116 WSUCF1.peg.2892 WSUCF1.peg.3036 | Oligopeptide transport ATP-binding protein OppF (TC 3.A.1.5.1) |
| WSUCF1.peg.117 WSUCF1.peg.2893 WSUCF1.peg.3035 | Oligopeptide transport ATP-binding protein OppD (TC 3.A.1.5.1) |
| WSUCF1.peg.118 WSUCF1.peg.3038 | Oligopeptide transport system permease protein OppC (TC 3.A.1.5.1) |
| WSUCF1.peg.119 WSUCF1.peg.1604 WSUCF1.peg.3037 | Oligopeptide transport system permease protein OppB (TC 3.A.1.5.1) |
| WSUCF1.peg.121 WSUCF1.peg.3039 | Oligopeptide ABC transporter, periplasmic oligopeptide-binding protein OppA (TC 3.A.1.5.1) |
| WSUCF1.peg.1396 | Subtilin biosynthesis sensor protein SpaK |
| WSUCF1.peg.2564 | Transcriptional regulator, TetR family |
| WSUCF1.peg.2983 | RNA-binding protein Hfq |
| WSUCF1.peg.3408 WSUCF1.peg.3409 | Autoinducer-2 production protein LuxS |
| WSUCF1.peg.3679 WSUCF1.peg.3680 WSUCF1.peg.3681 | Sensor protein ComP (EC 2.7.1.-) |
| WSUCF1.peg.3683 | Competence regulatory protein ComQ |

Table S10. Sporulation and natural competence

| Feature ID | Encoded protein |
| --- | --- |
| ***Sporulation*** |  |
| WSUCF1.peg.100 | Possible sporulation protein SpoIID precursor |
| WSUCF1.epg.177 | Sporulation protein YunB |
| WSUCF1.peg.210 | Stage II sporulation protein P |
| WSUCF1.peg.467 WSUCF1.peg.468 | Sporulation-specific protease YabG |
| WSUCF1.peg.479 | Fin: required for the switch from sigmaF to sigmaG during sporulation |
| WSUCF1.peg.492 | Stage II sporulation serine phosphatase for sigmaF activation (SpoIIE) |
| WSUCF1.peg.521 WSUCF1.peg.1515 | Sporulation kinase B |
| WSUCF1.peg.622 | Stage IV sporulation protein B |
| WSUCF1.peg.770 | Stage VI sporulation protein D |
| WSUCF1.peg.798 | Germination and sporulation protein GerM |
| WSUCF1.peg.802 | Major transcriptional regulator of spore coat formation GerE |
| WSUCF1.peg.835 | Sporulation protein SpoVIF (YjcA) |
| WSUCF1.peg.843 WSUCF1.peg.844 WSUCF1.peg.2023 | Stage V sporulation protein AC (SpoVAC) |
| WSUCF1.peg.845 WSUCF1.peg.2022 | Stage V sporulation protein AD (SpoVAD) |
| WSUCF1.peg.846 WSUCF1.peg.2021 | Stage V sporulation protein AE1 (SpoVAE1) |
| WSUCF1.peg.1084 | RNA polymerase sporulation specific sigma factor SigH |
| WSUCF1.peg.1384 WSUCF1.peg.3031 | Sporulation control protein |
| WSUCF1.peg.1474 | Protease synthase and sporulation negative regulatory protein PAI 2 |
| WSUCF1.peg.1531 | Stage II sporulation protein AA |
| WSUCF1.peg.1621 | Sporulation kinase A |
| WSUCF1.peg.1717 | Sporulation control protein Spo0M |
| WSUCF1.peg.2012 | Sporulation protein YhaL |
| WSUCF1.peg.2018 WSUCF1.peg.2455 | Stage V sporulation protein AF (SpoVAF) |
| WSUCF1.peg.2019 WSUCF1.peg.2020 | Stage V sporulation protein AE2 (SpoVAE2) |
| WSUCF1.peg.2024 WSUCF1.peg.2025 | Stage V sporulation protein AB (SpoVAB) |
| WSUCF1.peg.2026 | Stage V sporulation protein AA (SpoVAA) |
| WSUCF1.peg.2028 | RNA polymerase sporulation specific sigma factor SigF |
| WSUCF1.peg.2038 | Stage II sporulation protein M (SpoIIM) |
| WSUCF1.peg.2261 | Sporulation integral membrane protein YlbJ |
| WSUCF1.peg.2297 | Stage II sporulation protein Q (SpoIIQ) |
| WSUCF1.peg.2298 | Stage II sporulation protein D (SpoIID) |
| WSUCF1.peg.2467 | Stage II sporulation protein required for processing of pro-sigma-E (SpoIIR) |
| WSUCF1.peg.2467 | Stage II sporulation protein required for processing of pro-sigma-E (SpoIIR) |
| WSUCF1.peg.2515 WSUCF1.peg.3693 WSUCF1.peg.3694 | Sporulation integral membrane protein YtvI |
| WSUCF1.peg.2580 WSUCF1.peg.2581 | Sporulation sigma-E factor processing peptidase (SpoIIGA) |
| WSUCF1.peg.2582 | RNA polymerase sporulation specific sigma factor SigE |
| WSUCF1.peg.2583 | RNA polymerase sporulation specific sigma factor SigG |
| WSUCF1.peg.2621 | Sigma-G-dependent sporulation-specific SASP protein |
| WSUCF1.peg.2842 WSUCF1.peg.2843 WSUCF1.peg.2844 | Sporulation kinase E (EC 2.7.13.3) |
| WSUCF1.peg.2985 | Stage V sporulation protein SpoVK |
| WSUCF1.peg.3106 | KapD, inhibitor of KinA pathway to sporulation |
| WSUCF1.peg.3173 | Stage V sporulation protein B |
| WSUCF1.peg.3201 WSUCF1.peg.3202 | Stage IV sporulation pro-sigma-K processing enzyme (SpoIVFB) |
| WSUCF1.peg.3203 | Stage IV sporulation protein FA (SpoIVFA) |
| WSUCF1.peg.3254 | Stage III sporulation protein D |
| WSUCF1.peg.3507 WSUCF1.peg.3959 | Stage V sporulation protein D |
| WSUCF1.peg.3513 WSUCF1.peg.3514 | Stage V sporulation protein E |
| WSUCF1.peg.3533 WSUCF1.peg.3534 | Sporulation membrane protein YtrI |
| WSUCF1.peg.3657 | Stage II sporulation protein B |
| WSUCF1.peg.3861 | Sensor histidine kinase/sporulation kinase |
| WSUCF1.peg.3882 | Stage IV sporulation protein A |
| WSUCF1.peg.3930 | Sporulation protein cse60 |
| WSUCF1.peg.4009 | Sporulation kinase (EC 2.7.3.-) |
| WSUCF1.peg.4092 | Stage III sporulation protein AH |
| WSUCF1.peg.4093 | Stage III sporulation protein AG |
| WSUCF1.peg.4094 | Stage III sporulation protein AF |
| WSUCF1.peg.4095 | Stage III sporulation protein AE |
| WSUCF1.peg.4096 | Stage III sporulation protein AD |
| WSUCF1.peg.4097 | Stage III sporulation protein AC |
| WSUCF1.peg.4098 | Stage III sporulation protein AB |
| WSUCF1.peg.4099 WSUCF1.peg.4100 | Stage III sporulation protein AA |
| WSUCF1.peg.4181 | RNA polymerase sporulation specific sigma factor SigK |
| ***Natural competence*** |  |
| WSUCF1.peg.109 | Competence protein CoiA |
| WSUCF1.peg.282 | Late competence protein ComGA, access of DNA to ComEA |
| WSUCF1.peg.283 | Late competence protein ComGB, access of DNA to ComEA |
| WSUCF1.peg.284 | Late competence protein ComGC, access of DNA to ComEA, FIG007487 |
| WSUCF1.peg.285 | Late competence protein ComGD, access of DNA to ComEA, FIG012777 |
| WSUCF1.peg.286 | Late competence protein ComGE, FIG015564 |
| WSUCF1.peg.287 | Late competence protein ComGF, access of DNA to ComEA, FIG012620 |
| WSUCF1.peg.288 | Late competence protein ComGG, FIG028917 |
| WSUCF1.peg.1747 | Competence transcription factor |
| WSUCF1.peg.2532 | Late competence protein ComER, proline oxidase (EC 1.5.1.2) |
| WSUCF1.peg.2533 | Late competence protein ComEA, DNA receptor |
| WSUCF1.peg.2534 | dCMP deaminase (EC 3.5.4.12) @ late competence protein ComEB |
| WSUCF1.peg.2535 | DNA internalization-related competence protein ComEC/Rec2 |
| WSUCF1.peg.3477 | Competence protein ComF |
| WSUCF1.peg.3659 | Type IV pilus biogenesis protein PilO |
| WSUCF1.peg.3660 | Type IV pilus biogenesis protein PilN |
| WSUCF1.peg.3661 | Type IV pilus biogenesis protein PilM |

Table S11. Other regulators

| Feature ID | Encoded protein |
| --- | --- |
| WSUCF1.peg.39 | KinB signaling pathway activation protein |
| WSUCF1.peg.64 | Programmed cell death antitoxin YdcD |
| WSUCF1.peg.65 WSUCF1.peg.302 | Programmed cell death toxin YdcE |
| WSUCF1.peg.114 | Regulatory protein Spx |
| WSUCF1.peg.237 WSUCF1.peg.238 | Transcriptional repressor CcpN, MarR family |
| WSUCF1.peg.305 | RNA polymerase sigma factor SigB |
| WSUCF1.peg.371 | Programmed cell death antitoxin MazE |
| WSUCF1.peg.372 | Programmed cell death toxin MaxF |
| WSUCF1.peg.384 WSUCF1.peg.593 WSUCF1.peg.944 WSUCF1.peg.945 WSUCF1.peg.1644 WSUCF1.peg.2275 WSUCF1.peg.2344 WSUCF1.peg.2444 WSUCF1.peg.2565 WSUCF1.peg.3235 | Transcriptional regulator, AcrR family |
| WSUCF1.peg.410 | Phosphate regulon sensor protein PhoR |
| WSUCF1.peg.447 | Putative pheromone cAM373 precursor lipoprotein CamS |
| WSUCF1.peg.529 | Transcriptional repressor of the fructose operon, DeoR family |
| WSUCF1.peg.534 WSUCF1.peg.535 | Prevent host death protein, Phd antitoxin |
| WSUCF1.peg.536 | Death on curing protein, Doc toxin |
| WSUCF1.peg.605 | Two-component sensor histidine kinase Bsel_1486 |
| WSUCF1.peg.606 | Two-component response regulator Bsel_1485 |
| WSUCF1.peg.655 | Pyrophosphatase PpaX (EC 3.6.1.1) |
| WSUCF1.peg.658 | HPr kinase/phophorylase (EC 2.7.1.-) (EC 2.7.4.-) |
| WSUCF1.peg.696 WSUCF1.peg.3633 WSUCF1.peg.4056 | Transcriptional regulator, MerR family |
| WSUCF1.peg.739 | Two-component system YycFG regulatory protein YycI |
| WSUCF1.peg.740 | Two-component system YycFG regulatory protein YycH |
| WSUCF1.peg.741 | Two-component sensor kinase YycG |
| WSUCF1.peg.742 | Two-component response regulator YycF |
| WSUCF1.peg.800 | Uncharacterized transcriptional regulator YsmB, MarR family |
| WSUCF1.peg.825 WSUCF1.peg.1176 WSUCF1.peg.1373 | Sensor histidine kinase |
| WSUCF1.peg.917 | Lactate-responsive regulator LldR in Firmicutes, GntR family |
| WSUCF1.peg.1019 | Transcriptional repressor of arabinoside utilization operon, GntR family |
| WSUCF1.peg.1030 | Transcriptional repressor CzrA, ArsR family |
| WSUCF1.peg.1175 WSUCF1.peg.2479 | Uncharacterized transcriptional regulator YhgD, TetR family |
| WSUCF1.peg.1244 | Predicted transcriptional regulator of N-acetylglucosamine utilization, GntR family |
| WSUCF1.peg.1275 WSUCF1.peg.2068 | Positive regulator of CheA protein activity (CheW) |
| WSUCF1.peg.1276 | Signal transduction histidine kinase CheA (EC 2.7.3.-) |
| WSUCF1.peg.1305 | GTP-sensing transcriptional pleiotropic repressor CodY |
| WSUCF1.peg.1315 WSUCF1.pe.g1316 | Two-component response regulator |
| WSUCF1.peg.1326 | RNA polymerase sigma factor SigX |
| WSUCF1.peg.1472 WSUCF1.peg.1473 | Transcriptional regulator, GntR family domain/Aspartate aminotransferase (EC 2.6.1.1) |
| WSUCF1.peg.1482 WSUCF1.peg.1522 WSUCF1.peg.1564 WSUCF1.peg.1798 | Transcriptional regulator, GntR family |
| WSUCF1.peg.1616 | Phosphotransferase system, phosphocarrier protein HPr |
| WSUCF1.peg.1687 | Alkaline phosphatase synthesis transcriptional regulatory protein PhoP |
| WSUCF1.peg.1722 | Transcriptional regulator, ArsR family |
| WSUCF1.peg.1778 WSUCF1.peg.2443 | Transcriptional regulator, AraC family |
| WSUCF1.peg.1785 | Maltose operon transcriptional repressor MalR, LacI family |
| WSUCF1.peg.1791 | Two component system histidine kinase |
| WSUCF1.peg.1812 | Response regulator of zinc sigma-54-dependent two-component system |
| WSUCF1.peg.1826 | LysR-family transcriptional activator CysL |
| WSUCF1.peg.2060 | Two-component response regulator BceR |
| WSUCF1.peg.2061 WSUCF1.peg.2062 | Two-component sensor histidine kinase BceS |
| WSUCF1.peg.2063 | Bacitracin export ATP-binding protein BceA |
| WSUCF1.peg.2064 | Bacitracin export permease protein BceB |
| WSUCF1.peg.2081 | Probable HTH-type transcriptional regulator endR |
| WSUCF1.peg.2216 WSUCF1.peg.2912 | Transcriptional regulator, LacI family |
| WSUCF1.peg.2293 WSUCF1.peg.3047 | DNA-binding response regulator, AraC family |
| WSUCF1.peg.2294 WSUCF1.peg.2683 | Two-component sensor histidine kinase YesM |
| WSUCF1.peg.2421 | Fatty acid metabolism regulator protein FadR, TetR family |
| WSUCF1.peg.2432 | Signal transduction histidine kinase |
| WSUCF1.peg.2684 | Two-component response regulator YesN |
| WSUCF1.peg.2627 | Transmembrane histidine kinase CsrS |
| WSUCF1.peg.2724 | HTH-type transcriptional regulator tnrA |
| WSUCF1.peg.2966 | Serine/threonine protein kinase PrkC |
| WSUCF1.peg.2967 | Protein serine/threonine phosphatase PrpC |
| WSUCF1.peg.3100 | Transcriptional regulator, AsnC family |
| WSUCF1.peg.3360 | Catabolite repression HPr-like protein Crh |
| WSUCF1.peg.3393 | Galactose operon repressor, GalR-LacI family of transcriptional regulators |
| WSUCF1.peg.3798 | Protease production regulatory protein HPr (ScoC) |

Table S12. Defense system

| Feature ID | Encoded protein |
| --- | --- |
| ***Multidrug resistance*** |  |
| WSUCF1.peg.369 | Bacitracin transport permease protein bcrB |
| WSUCF1.peg.1052 WSUCF1.peg.4010 | Multi antimicrobial extrusion protein (Na(+)/drug antiporter), MATE family of MDR efflux pumps |
| WSUCF1.peg.1169 WSUCF1.peg.1170 | Multidrug resistance protein ErmB |
| WSUCF1.peg.1183 | RND multidrug efflux transporter, Acriflavin resistance protein |
| WSUCF1.peg.1184 | TetR family transcriptional regulator probably coupled to RND multidrug efflux transporter |
| WSUCF1.peg.1211 | Penicillin-binding protein 4B |
| WSUCF1.peg.1581 | Penicillin-binding protein 3 |
| WSUCF1.peg.2613 WSUCF1.peg.2614 | Penicillin-binding protein 1A/1B |
| WSUCF1.peg.1516 | Fosmidomycin resistance protein |
| WSUCF1.peg.1949 WSUCF1.peg.2995 WSUCF1.peg.3024 WSUCF1.peg.3083 | Permease of the drug/metabolite transporter (DMT) superfamily |
| WSUCF1.peg.2882 | Daunorubicin resistance transmembrane protein |
| WSUCF1.peg.2883 WSUCF1.peg.3629 | Daunorubicin resistance ATP-binding protein drrA |
| ***CRISPR-Cas system*** | |
| WSUCF1.peg.3340 WSUCF1.peg.3341 | CRISPR-associated protein TM1812 |
| WSUCF1.peg.3342 | CRISPR-associated RAMP Cmr1 |
| WSUCF1.peg.3343 WSUCF1.peg.3344 | CRISPR-associated RAMP Cmr2 |
| WSUCF1.peg.3345 | CRISPR-associated RAMP Cmr3 |
| WSUCF1.peg.3346 | CRISPR-associated RAMP Cmr4 |
| WSUCF1.peg.3347 | CRISPR-associated RAMP Cmr5 |
| WSUCF1.peg.3348 | CRISPR-associated RAMP Cmr6 |
| WSUCF1.peg.4159 | CRISPR-associated endoribonuclease Cas6 |
| ***Restriction-modification (R-M) system*** | |
| WSUCF1.peg.596 WSUCF1.peg.700 WSUCF1.peg.2357 WSUCF1.peg.3894 | Type III restriction-modification system methylation subunit (EC 2.1.1.72) |
| WSUCF1.peg.1726 WSUCF1.peg.2206 | Type I restriction-modification system, restriction subunit R (EC 3.1.21.3) |
| WSUCF1.peg.1727 | Type I restriction-modification system, specificity subunit S |
| WSUCF1.peg.1728 | Type I restriction-modification system, DNA-methyltransferase subunit M (EC 2.1.1.72) |
| WSUCF1.peg.3601 | Type III restriction-modification system restriction subunit (EC 3.1.21.5) |
| ***Other defense proteins*** | |
| WSUCF1.peg.992 | Immunity protein SdpI |
| WSUCF1.peg.1393 WSUCF1.peg.1394 | Lantibiotic ABC transporter |
| WSUCF1.peg.1395 | Lantibiotic biosynthesis protein |





Figure S1. Synthesis of lipid accepter through MEP/DOXP pathway in *Geobacillus* sp. WSUCF1 inferred from genomic sequence data


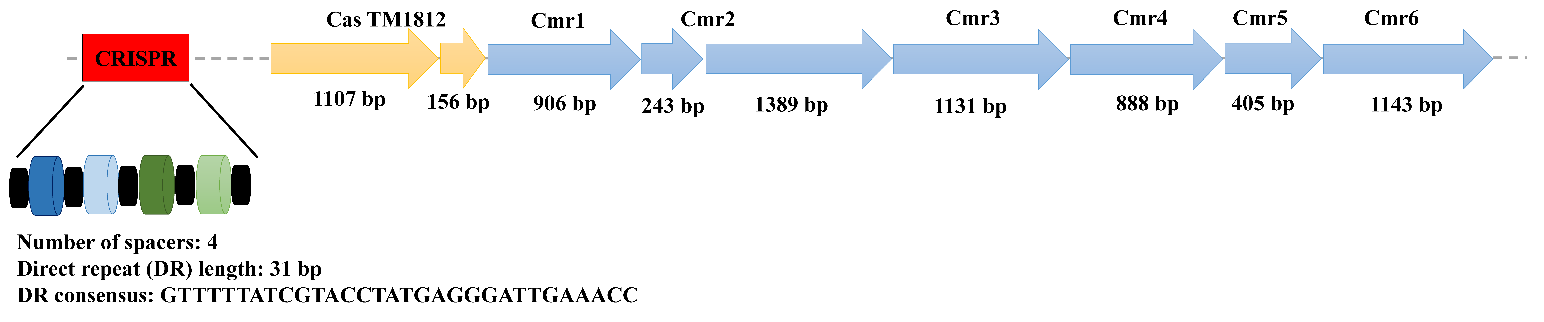


Figure S2. Structure of CRISPR-Cas locus in *Geobacillus* sp. WSUCF1. The direct repeats are black color-coded, and the spacers are other color-coded.


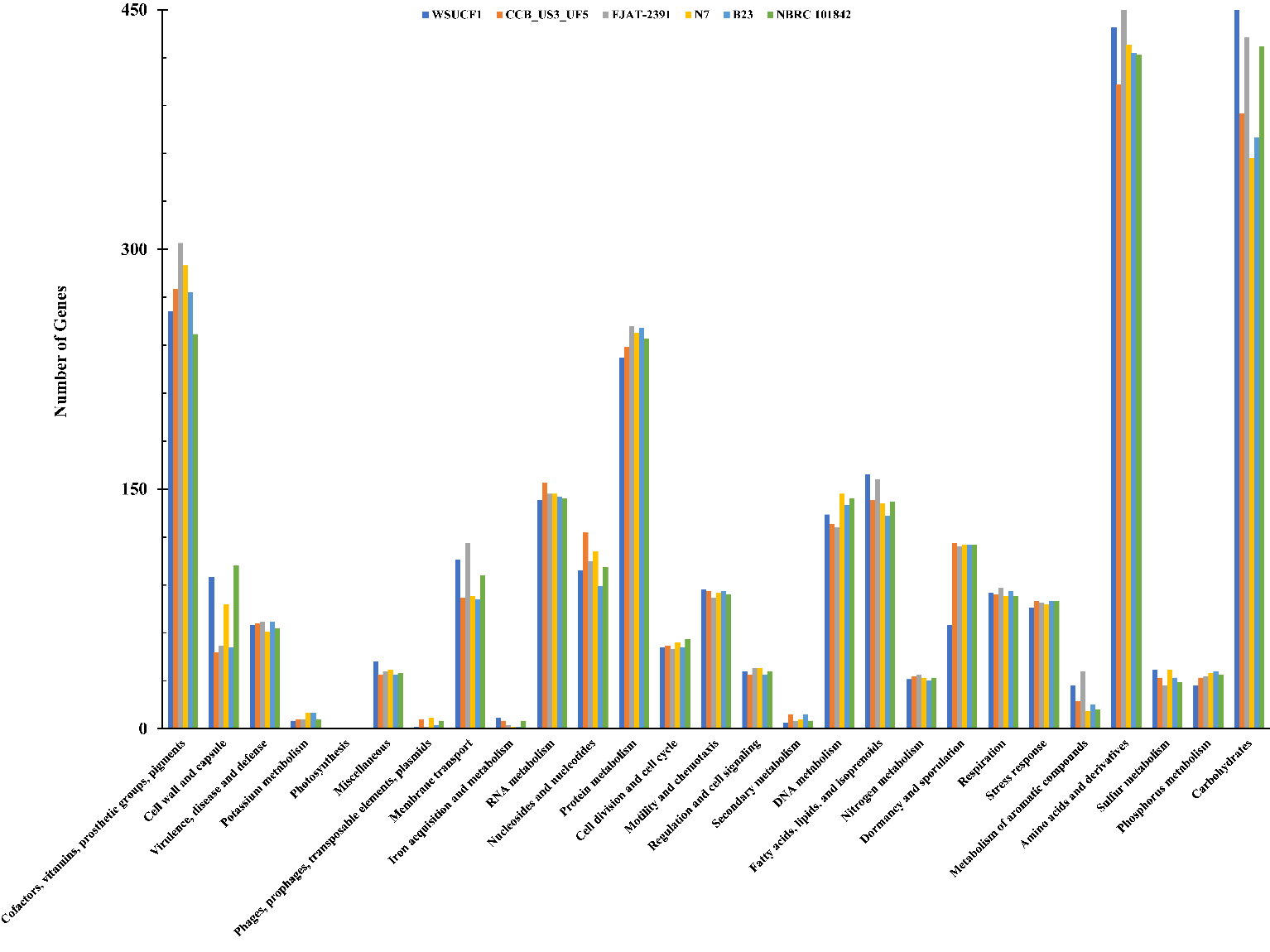


Figure S3. Comparison of subsystem feature of WSUCF1 and other five sequenced *Geobacillus* spp. according to RAST server classification

**Reference**

1 Aziz, R. K. *et al.* The RAST server: Rapid annotations using subsystems technology. *BMC Genomics* **9**, 75, doi:10.1186/1471-2164-9-75 (2008).

2 Boguslawski, K. M., Hill, P. A. & Griffith, K. L. Novel mechanisms of controlling the activities of the transcription factors Spo0A and ComA by the plasmid-encoded quorum sensing regulators Rap60-Phr60 in *Bacillus subtilis*. *Mol. Microbiol.* **96**, 325-348, doi:10.1111/mmi.12939 (2015).

3 Sarilmiser, H. K., Ates, O., Ozdemir, G., Arga, K. Y. & Toksoy Oner, E. Effective stimulating factors for microbial levan production by *Halomonas smyrnensis* AAD6T. *J. Biosci. Bioeng.* **119**, 455-463 (2015).

4 Slamti, L. *et al.* CodY regulates the activity of the virulence quorum sensor PlcR by controlling the import of the signaling peptide PapR in *Bacillus thuringiensis*. *Front. Microbiol.* **6**, doi:10.3389/fmicb.2015.01501 (2016).

5 Tu, K. C., Waters, C. M., Svenningsen, S. L. & Bassler, B. L. A small-RNA-mediated negative feedback loop controls quorum-sensing dynamics in *Vibrio harveyi*. *Mol. Microbiol.* **70**, 896-907, doi:10.1111/j.1365-2958.2008.06452.x (2008).

6 van Kessel, J. C., Ulrich, L. E., Zhulin, I. B. & Bassler, B. L. Analysis of activator and repressor functions reveals the requirements for transcriptional control by LuxR, the master regulator of quorum sensing in *Vibrio harveyi*. *MBio* **4**, doi:10.1128/mBio.00378-13 (2013).

7 Ramos, J. L. *et al.* The TetR family of transcriptional repressors. *Microbiol. Mol. Biol. Rev.* **69**, 326-356 (2005).

8 Lenz, D. H. *et al.* The small RNA chaperone Hfq and multiple small RNAs control quorum sensing in *Vibrio harveyi* and *Vibrio cholerae*. *Cell* **118**, 69-82 (2004).

9 Su, F. & Xu, P. Genomic analysis of thermophilic *Bacillus* coagulans strains: Efficient producers for platform bio-chemicals. *Sci. Rep.* **4**, 3926, doi:10.1038/srep03926 (2014).

10 Rai, N., Rai, R. & Venkatesh, K. V. in *Quorum Sensing vs Quorum Quenching: A Battle with No End in Sight* (ed Vipin Chandra Kalia) 61-64 (Springer India, 2015).

11 Wu, Q., Tun, H. M., Leung, F. C.-C. & Shah, N. P. Genomic insights into high exopolysaccharide-producing dairy starter bacterium *Streptococcus thermophilus* ASCC 1275. *Sci. Rep.* **4**, 4974, doi:10.1038/srep04974 (2014).

12 Vickerman, M. M. *et al.* A genetic determinant in *Streptococcus gordonii* Challis encodes a peptide with activity similar to that of enterococcal sex pheromone cAM373, which facilitates intergeneric DNA transfer. *J. Bacteriol.* **192**, 2535-2545, doi:10.1128/JB.01689-09 (2010).

13 Claverys, J.-P., Prudhomme, M. & Martin, B. Induction of competence regulons as a general response to stress in Gram-positive bacteria. *Annu. Rev. Microbiol.* **60**, 451-475, doi:10.1146/annurev.micro.60.080805.142139 (2006).

14 César, C. E. *et al.* Unconventional lateral gene transfer in extreme thermophilic bacteria. *Int. Microbiol.* **14**, 187-199 (2011).

15 Magiorakos, A. P. *et al.* Multidrug‐resistant, extensively drug‐resistant and pandrug‐resistant bacteria: An international expert proposal for interim standard definitions for acquired resistance. *Clin. Microbiol. Infect.* **18**, 268-281, doi:10.1111/j.1469-0691.2011.03570.x (2011).

16 Piddock, L. J. V. Multidrug-resistance efflux pumps? Not just for resistance. *Nat. Rev. Microbiol.* **4**, 629, doi:10.1038/nrmicro1464 (2006).

17 Studholme, D. J. Some (bacilli) like it hot: Genomics of *Geobacillus* species. *Microb. Biotechnol.* **8**, 40-48, doi:10.1111/1751-7915.12161 (2015).

18 Koonin, E. V. & Wolf, Y. I. Evolution of the CRISPR-Cas adaptive immunity systems in prokaryotes: Models and observations on virus-host coevolution. *Mol. Biosyst.* **11**, 20-27, doi:10.1039/C4MB00438H (2015).

19 Mojica, F. J. M., Díez-Villaseñor, C., García-Martínez, J. & Almendros, C. Short motif sequences determine the targets of the prokaryotic CRISPR defence system. *Microbiology* **155**, 733-740, doi:doi:10.1099/mic.0.023960-0 (2009).

20 Sorek, R., Kunin, V. & Hugenholtz, P. CRISPR — a widespread system that provides acquired resistance against phages in bacteria and archaea. *Nat. Rev. Microbiol.* **6**, 181, doi:10.1038/nrmicro1793 (2008).

21 Chellapandi, P. & Ranjani, J. Knowledge-based discovery for designing CRISPR-CAS systems against invading mobilomes in thermophiles. *Syst. Synth. Biol.* **9**, 97-106, doi:10.1007/s11693-015-9176-8 (2015).

22 Gophna, U. *et al.* No evidence of inhibition of horizontal gene transfer by CRISPR–Cas on evolutionary timescales. *ISME J.* **9**, 2021-2027, doi:10.1038/ismej.2015.20 (2015).

23 Chen, F., Gao, Y., Chen, X., Yu, Z. & Li, X. Quorum quenching enzymes and their application in degrading signal molecules to block quorum sensing-dependent infection. *Int. J. Mol. Sci.* **14**, 17477-17500, doi:10.3390/ijms140917477 (2013).

24 Scarascia, G., Wang, T. & Hong, P.-Y. Quorum sensing and the use of quorum quenchers as natural biocides to inhibit sulfate-reducing bacteria. *Antibiotics* **5**, 39, doi:10.3390/antibiotics5040039 (2016).

25 Seo, M.-J., Lee, B.-S., Pyun, Y.-R. & Park, H. Isolation and characterization of N-acylhomoserine lactonase from the thermophilic bacterium, *Geobacillus caldoxylosilyticus* YS-8. *Biosci. Biotechnol. Biochem.* **75**, 1789-1795, doi:10.1271/bbb.110322 (2011).

26 Wright, Addison V., Nuñez, James K. & Doudna, Jennifer A. Biology and applications of CRISPR systems: Harnessing nature’s toolbox for genome engineering. *Cell* **164**, 29-44 (2016).

27 Harrington, L. B. *et al.* A thermostable Cas9 with increased lifetime in human plasma. *Nat. Commun.* **8**, 1424, doi:10.1038/s41467-017-01408-4 (2017).

28 Nakano, C., Ozawa, H., Akanuma, G., Funa, N. & Horinouchi, S. Biosynthesis of aliphatic polyketides by type III polyketide synthase and methyltransferase in *Bacillus subtilis*. *J. Bacteriol.* **191**, 4916-4923 (2009).

29 Yu, D., Xu, F., Zeng, J. & Zhan, J. Type III polyketide synthases in natural product biosynthesis. *IUBMB Life* **64**, 285-295, doi:10.1002/iub.1005 (2012).

30 Rastogi, G. *et al.* Characterization of thermostable cellulases produced by *Bacillus* and *Geobacillus* strains. *Bioresour. Technol.* **101**, 8798-8806, doi:10.1016/j.biortech.2010.06.001 (2010).

31 Aliyu, H., Lebre, P., Blom, J., Cowan, D. & De Maayer, P. Phylogenomic re-assessment of the thermophilic genus *Geobacillus*. *Syst. Appl. Microbiol.* **39**, 527-533, doi:10.1016/j.syapm.2016.09.004 (2016).

32 Burgess, S. A., Flint, S. H., Lindsay, D., Cox, M. P. & Biggs, P. J. Insights into the *Geobacillus stearothermophilus* species based on phylogenomic principles. *BMC Microbiol.* **17**, 140, doi:10.1186/s12866-017-1047-x (2017).

1. * Corresponding authors

   *E-mail addresses:* David.Salem@sdsmt.edu (D. R. Salem) and Rajesh.Sani@sdsmt.edu (R. K. Sani) [↑](#footnote-ref-1)
